# A longitudinal blended learning curriculum for bedside ultrasound education in pulmonary and critical care fellowship

**DOI:** 10.1186/s12909-024-06584-8

**Published:** 2025-01-24

**Authors:** Harry Kuperstein, Kunal Gada, Werda Alam, Sahar Ahmad

**Affiliations:** 1 Renaissance School of Medicine at Stony Brook University, Stony Brook, NY USA; 2 StatCare Pulmonary, Knoxville, TN USA; 3 Mount Sinai West, New York, NY USA; 4 Division of Pulmonary, Critical Care and Sleep Medicine, Department of Medicine, Renaissance School of Medicine at Stony Brook University, Stony Brook, NY USA

**Keywords:** Ultrasound education, Bedside ultrasound, Ultrasonography, Blended learning, Asynchronous learning, Critical care medicine, Pulmonary and critical care medicine

## Abstract

**Background:**

There exists no standardized longitudinal curriculum for teaching bedside ultrasonography (US) in Pulmonary and Critical Care Medicine (PCCM) fellowship programs. Given the importance of mastering bedside US in clinical practice, we developed an integrated year-long US curriculum for first-year PCCM fellows.

**Methods:**

11 first-year PCCM fellows completed the entire seven-step Blended Learning Curriculum. We provide results from an evaluation of Step I, the initial training course. Evaluation included a 17-question multiple-choice knowledge test and a hands-on skill exam delivered pre-, immediately post-, and 6 months post-course. Performance on these same evaluation measures was compared between learners who completed a traditionally designed curriculum, which contained a formal in-person didactic course, and learners who completed a blended learning curriculum covering the same learning objectives.

**Results:**

All learners showed a significant improvement immediately after the course in both knowledge (*p* = 0.007) and skills (*p* = 0.004) with adequate retention of both knowledge and skills after 6 months. Scores on a multiple-choice knowledge test increased from a median (interquartile range [IQR]) of 24% (15–41%) pre-course to a median of 71% (59–82%) post-course, while scores on a hands-on skill exam increased from a median of 16% (7–45%) pre-course to a median of 87% (74–94%) post-course. There was no difference in learning or retention between those who learned via the blended learning model as compared with a more traditional model. Learners agreed the course was well-designed, with relevant learning topics, sufficient time to learn, and fair evaluation modalities. The blended learning model required 15 fewer faculty-hours than the traditional learning model.

**Discussion:**

A blended learning model for bedside US education implemented at a single PCCM fellowship performs comparably to a traditional model for both acquisition and retention of knowledge and skills. The incorporation of asynchronous learning mitigates the barrier of insufficient time and quantity of US skilled teaching faculty that many PCCM fellowships face and provides flexibility to both instructors and learners.

**Supplementary Information:**

The online version contains supplementary material available at 10.1186/s12909-024-06584-8.

## Introduction

Bedside ultrasonography (US) is invaluable to the care of patients encountered in Pulmonary and Critical Care Medicine (PCCM) practice and training in US is required by the Accreditation Council for Graduate Medical Education (ACGME) [1]. However, no proven curriculum or paradigm exists for US training at the PCCM fellowship level.

Barriers to US training at the PCCM fellowship level include content redundancy, loss of trainee motivation, lack of formal assessment, and time limitations of both trainees and faculty [2–4]. Using a standardized curriculum, content redundancy can be eliminated. An effective bedside US training pedagogy will need to engage internal motivations of adult learners. In the case of PCCM trainees, clinical application for patient care is the expected internal motivation, so it follows that US training for this learner group should include an emphasis on clinical applications of the learned skills with patient and case-based interactions included [5]. Formal assessment can be incorporated into curricular activities. The lattermost constraint, time limitations on trained faculty, can be addressed by a curriculum that alleviates the need for live didactics or evaluation requirements. This particular concern of having sufficiently skilled and available faculty has been reported in varied clinical settings for over a decade; in 2010, 41% of PCCM and critical care medicine (CCM) program directors believed there was not a sufficient amount of trained faculty for US instruction [2], while in 2020, 48% of chiefs from Veterans Affairs centers with an intensive care unit (ICU) believed a lack of trained providers was a barrier to training [6]. 

PCCM program directors and US educators have attempted several different models for US education. One model incorporated a regional three-day US course jointly led by faculty at neighboring institutions which incorporated blended learning didactics, but did not contain longitudinal follow-up and required scheduling coordination between several fellowship programs [7]. Another model for US training for critical care physicians describes a two-day, seven-hour workshop consisting of in-person didactics and practical stations [8]. Limitations described with this particular workshop were consistent with the aforementioned barriers, including a need for five faculty instructors per session and a desire for more self-study material for continued learning beyond the workshop. Another study compared two randomly assigned groups of CCM fellows into a standard or intensive training group, where the intensive group received an additional eight hour training session [9]. While both groups in this study had significantly improved from the start of a six-month training period, this additional discrete training session did not lead to stronger performance than those who did not receive it. A 2016 systematic review of published curricula specific to cardiac critical care US noted that hybrid methods seem most efficient, but added that many studies of these curricula failed to assess baseline US knowledge and skills and that maintenance of skill was not well-assessed within the selected studies [10]. A more recent model for US training described a longitudinal curriculum taught by four faculty members leading weekly in-person scanning sessions, made possible due to adequate involvement of trained US educators but only generalizable to institutions with similar numbers of trained faculty [11]. 

Generally, attempts have been made to incorporate teaching modalities beyond in-person instruction. One program attempted remote US training with wireless archiving and offline oversight [12]. This effort made no comparison between traditional didactics and this asynchronous method. A separate study incorporated blended didactics and bedside sessions into a six-week course for 8 PCCM fellows with improvements in image acquisition and subject knowledge [13]. However, this course did not extend longitudinally through the first year of fellowship and did not assess retention or effectiveness over multiple years of training. Additionally, other studies have shown effectiveness of other teaching modalities: online didactics improved bedside US image recognition [14], proctored instruction was effective for teaching image acquisition [15], simulation training was used successfully for US-guided procedural training [16], and the combination of multimedia and proctored hands-on instruction has been implemented with success in echocardiography training [17]. 

Incorporating many of the above proven methods of US training, we aimed to develop and implement a year-long US training curriculum for PCCM fellows utilizing a mixed-modality blended learning format. Our program emphasized practical US application for the PCCM practitioner and aimed for the development of a proficient clinician-sonographer. We hypothesize this program will result in learning outcomes comparable with a traditional curriculum composed of in-person instruction as described in the literature but with less faculty-hours required and with adequate learner satisfaction. Our primary endpoint was a statistically significant improvement in medical knowledge and hands-on skills. A secondary endpoint was learner satisfaction measured quantitatively on a course evaluation.

## Methods

### Curriculum development

A formal program for US education has been a staple of our institution since 2013, which was created using the American College of Chest Physicians (ACCP) statement as a blueprint for learning objectives [18]. A longitudinal, year-long curriculum incorporating blended learning for bedside US instruction was introduced in 2017, referred to herein as the “Blended Learning Curriculum.” Implementation and evaluation of this curriculum was approved by the Institutional Review Board at Stony Brook University Hospital (IRB #2014–2657). Target learners were first-year PCCM fellows with varying degrees of bedside US training; no pre-requisite knowledge was assumed at the initiation of the program. Instructors were PCCM attending physicians with formal US training either through fellowship or through completion of a formal national US training course. All hands-on teaching and independent ultrasound capture was performed using a portable US unit approved by the institution for patient care.

### Traditional curriculum

The Blended Learning Curriculum modified an existing longitudinal curriculum for bedside US, referred to herein as the “Traditional Curriculum,” used between 2013 and 2016. The Blended Learning Curriculum, which incorporates asynchronous learning and reduces in-person faculty hours was conceived in response to faculty and fellows’ feedback pertaining to the Traditional Curriculum which included that faculty reported difficult-to-sustain level of in-person commitment, and fellows reported that more time was needed for didactics. The Blended Learning Curriculum not only reduces the personal time commitment of the faculty but also provides asynchronous didactics whereby the learner can control the time spent on didactics.

The traditional model deviated from the blended learning model specifically in relation to Step I of the curriculum, composed of formal coursework. The Traditional Curriculum consisted of five strictly in-person teaching sessions lasting four hours each. Similarly, the Blended Learning Curriculum included five mixed-method teaching blocks with material constituting four hours of work each, but omitted most in-person didactics, some in-person skills sessions and in-person case discussions in favor of a mostly asynchronous experience, thus relieving much of the required in-person faculty time. To determine the extent of time saved, instructor hours for each course format, the Traditional Curriculum and the Blended Learning Curriculum, were recorded.

### New blended learning curriculum

The Blended Learning Curriculum integrated a stepwise paradigm spanning the first year of fellowship (Table 1). Learning objectives, material covered, and course evaluations aligned with the American College of Chest Physicians consensus statement on critical care ultrasonography training [18]. 

**Table 1:** Blended learning curriculum paradigm

Step	Timeframe of Application	Activities
Step I: Formal Coursework	Month 1	Coursework consisted of asynchronous learning by way of the SonoSim platform education tool, with limited in-person didactic style modules and simulations paired with hands-on teaching by educational faculty. Pre-reading assignments included selections from literature, textbook chapters, and online resources; in addition, for the Blended Learning Curriculum, asynchronous learning modules are outlined (see Additional file 1).
Step II: Portfolio Development	Month 2–12	Each trainee developed and maintained image sets in digital portfolios according to a mandatory list and the cases they participate in. Portfolio development was divided into four levels of independent image acquisition in the medical ICU (see Additional file 2).
Step III: Deliberate Practice Session	Month 6	Trainees participated in a two-hour, individualized learner and instructor session including review of self-perceived strengths and weaknesses, review of image portfolio and a skills practical, the contents of which are provided (see Additional file 3).
Step IV: Portfolio Maintenance	Month 2–12 with case presentations scheduled quarterly	Fellows presented a new or ongoing case and discussed the ultrasound images and techniques used to manage the patient. This was followed by a short didactic on a topic relevant to the case.
Step V: Clinically Targeted Sessions	Month 2–12 with educational events scheduled quarterly	Fellows participated in group scanning sessions, targeted towards combining US imaging findings clinical context. Sessions included US rounds in the medical ICU, US consultation services on medicine, medical ICU and pulmonary teams, US electives, US during and after cardiac arrest events, case-based sessions, patients with dyspnea, patients with undifferentiated shock, and conducting an IVC evaluation for fluid responsiveness.
Step VI: Training the Trainer	Month 6–12 with educational events occurring once or twice	Fellows participated in train the trainer sessions, patient care sessions in which the trainee guides the trainer. A trainee taught at the student, resident, and junior fellow level under supervision of the trainer, and a provided guide for the trainee is provided (see Additional file 4).
Step VII: Final Competency Testing and Review	End of final year of PCCM Fellowship	Prior to program graduation, core bedside US skills and knowledge were tested in objective structured clinical examination (OSCE) format. Case introduction is provided in a case file (see Additional file 5), the corresponding videos are provided separately (see Additional file 6), and the answer key is provided (see Additional file 7).

**Table 2:** Sessions and topics of Step I of blended learning curriculum

Session	Sessions	Notes
1	Introduction to Critical Care Ultrasound	Utility and scope
2	Fundamentals	Ultrasound physics, machine settings, probe selection
3	Vascular Access and Diagnostics	Upper extremity vascular access (axillary radial artery, cephalic vein, brachial vein, basilic vein); deep venous thrombosis study; femoral vein and artery access; internal jugular vein access; subclavian vein access
4	Chest Ultrasound	Lungs, pleura, diaphragm, airway: clinical applications; related clinical applications and literature
5	Abdomen and Retroperitoneum	Evaluation for hemoperitoneum and pneumoperitoneum; great vessels anatomy; kidney; related clinical applications and literature
6	Inferior vena cava (IVC)	IVC ultrasound; related clinical applications and literature
7	Echocardiography I	Basic echocardiography; related clinical applications and literature
8	Echocardiography II	Advanced echocardiography
9	Ultrasound Protocols and Clinical Use	Case-based review of ultrasound protocols for clinical applications: central venous access; undifferentiated shock; respiratory failure; pulmonary edema; hemoperitoneum; cardiac arrest

### Step 1 blended learning curriculum

Step 1 of the Blended Learning Curriculum included five didactic days (constituting nine sessions total) with associated SonoSim modules and in-person didactic time (see Additional file 1). The nine sessions covered US fundamentals, vascular access, pulmonary US, abdominal and extremity US, inferior vena cava (IVC) measurement, echocardiography, and critical care ultrasound (Table 2). Mandatory independent module time was approximately 2 h and 30 min for the first day’s sessions and under an hour for each subsequent day’s sessions. Each session contained specific scanning objectives to be achieved either by virtual simulation on the SonoSim platform or by in-person practice. Additional self-directed learning resources, which were optional for participants, included online resources, two textbook chapters and several primary literature sources.

### Evaluation

Evaluation of both curricula models included medical knowledge testing, hands-on skills testing, and course evaluation feedback from learners. Assessments were conducted before the course (Pre-Course), immediately after the course (Post-Course) and six months following the completion of the course (6-Month Retention). The 17-question written knowledge exam covered content related to US physics, US image recognition and interpreting US images within clinical context and is provided (see Additional file 8). Specific learning domains covered by the written knowledge exam included cardiac US, pleural US, abdominal US and vascular structure identification. This assessment has been internally validated in a population of internal medicine resident learners, who scored an average of 39% when administered before a five-day US elective and 66% following this US elective. The bedside skill exam conducted during Step III covered content related to image acquisition, US machine operation, anatomical knowledge and clinical correlation of findings (see Additional file 3). Specific learning domains covered during the bedside skill exam included identifying a site for internal jugular venous access, performing a deep venous thrombosis study, performing pleural US, performing abdominal US, obtaining four echocardiography windows and measuring inferior vena cava diameter. This bedside skill exam was observed and graded by the same US educator to maintain reliability between examinations. It has not been previously validated but covers all aspects of ACGME-mandated US indications [1]. We note that our skills exam was developed and used prior to the publication of a validated assessment tool and remained in continuous use during the study period to enable longitudinal comparison [19]. 

### Statistical analysis

Our study is a retrospective evaluation of routinely collected assessment and course evaluation data, so no sample size or power analysis was conducted a priori. Making comparison to a prior prospective US training study referenced within this report which had conducted power analyses, our sample sizes and assessment methods are similar [9]. Non-parametric methods were utilized for intra-curricular analysis as no assumption of normality could be assumed within the data. A Wilcoxon signed rank test was used to compare changes in individual scores within each curriculum. Statistical significance was assessed at the 0.05 level.

For inter-curricular analysis, paired methods could not be utilized. A Mann-Whitney test was used to assess for a change in score between the two curricula for pre-, post-test and 6-month retention scores, assessing statistical significance at the 0.05 level. Statistical analysis was conducted using Microsoft Excel and SAS.

Faculty-hours were calculated by number of instructors multiplied by hours required. All data are otherwise reported as median value with the interquartile range. Any missing data was due to trainee or instructor time constraints and is noted in alongside results where applicable.

## Results

11 first-year PCCM fellows participated in the year-long Blended Learning Curriculum in 2017 and 2018. Demographic details were collected for 9 of these 11 first-year PCCM fellows at the start of the Blended Learning Curriculum. 1 fellow had no experience with critical care US, 2 fellows had less than 1 year of experience, and 6 fellows had 3–5 years. 1 fellow had not completed any US training, 2 fellows had only done self-directed learning, 2 fellows had some form of supervised learning, 1 fellow had completed a formal course without supervised training while 3 fellows had completed a formal course with supervised training. Participants’ confidence in clinical use of critical care US was also surveyed at this time (Table 3).

**Table 3:** Pre-course confidence for blended learning curriculum learners. 9 PCCM fellows provided survey responses to prompts relating to their confidence in aspects of critical care POCUS. All prompts related to learning objectives of the curriculum. Options provided were “Strongly agree,” “Agree,” “Neutral,” “Disagree,” and “Strongly disagree.” Further demographic information on learners’ experience with POCUS is provided in the main text

Prompt	Strongly Agree	Agree	Neutral	Disagree	Strongly Disagree
I am confident in the management of shock and hypotension using critical care ultrasound technique.	2	2	1	2	2
I am confident in my skill of acquiring a dynamic sonographic image of the IVC at the patient’s bedside.	2	2	2	1	2
I am confident in the management of pulmonary edema using critical care ultrasound technique.	3	1	1	3	1
I am confident in my skill of acquiring images of the lung parenchyma at the patient’s bedside.	3	1	2	2	1

**Fig. 1:**
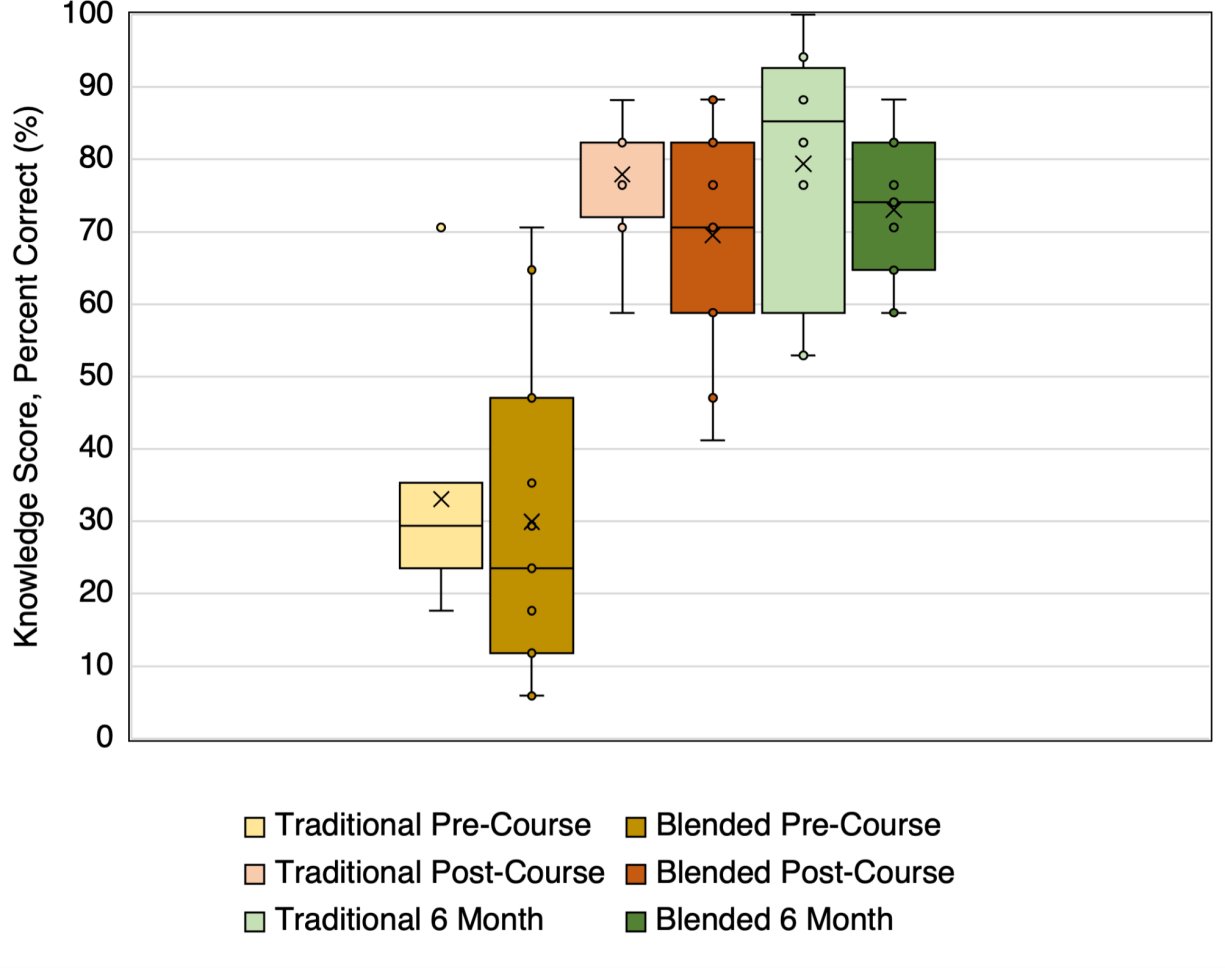
Median knowledge scores at pre-course, post-course and 6 month timepoints. Scores on a 17-question multiple choice knowledge exam are provided from participants in the Traditional Curriculum course (n = 8) and the Blended Learning Curriculum (n = 11) at pre-course, post-course and six month follow-up time points. Data is reported with median as indicated by the horizontal line, interquartile range as shown with the shaded box, and mean as indicated by the X marker. Asterisk (*) indicates statistical significance at the 0.05 level as compared to the pre-course score within each curriculum. One outlier exists within the Traditional Pre-Course data for a fellow who scored 71% on the knowledge exam without as significant of a corresponding difference on the Pre-Course data for the skills exam (37%) thus representing stronger medical knowledge upon starting the course without as strong hands-on skills.

Participants’ clinical US knowledge was assessed using a written knowledge exam before the course, immediately after the course and six months post-course (Fig. 1). Comparison was made to results of 8 fellows from prior years’ Traditional Curriculum. Pre-course and post-course assessments were completed during Step 1 of each curriculum.

**Fig. 2:**
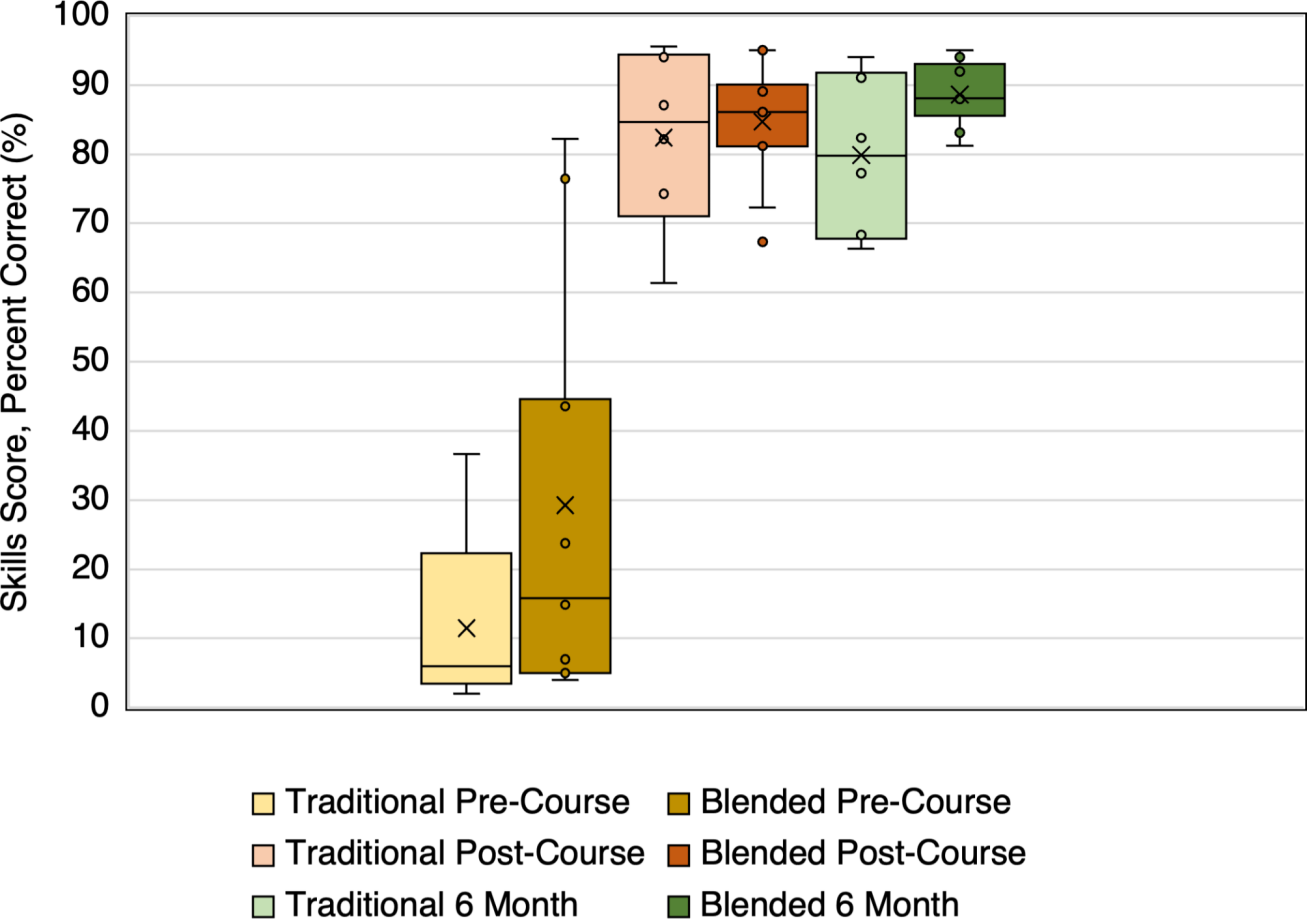
Median skill exam scores at pre-course, post-course and 6 month timepoints. Scores on a hands-on skill exam are provided from participants in the Traditional Curriculum course (n = 5) and the Blended Learning Curriculum (n = 9) at pre-course, post-course and six month follow-up time points. Data is reported with median as indicated by the horizontal line, interquartile range as shown with the shaded box, and mean as indicated by the X marker. Asterisk (*) indicates statistical significance at the 0.05 level as compared to the pre-course score within each curriculum.

Clinical US skills were assessed using a bedside skill exam at the same timepoints (Fig. 2). 2 of the 11 participating PCCM fellows did not complete skill exams at all three timepoints and were excluded from analysis. Comparison was made to results of 5 fellows from prior years’ Traditional Curriculum as 3 of 8 participating PCCM fellows did not complete skill exams at all three timepoints and were similarly excluded from analysis.

For the written knowledge exam, the median (IQR) post-course score of 71% (59–82%) was significantly higher than the pre-course score of 24% (15–41%) (*p* = 0.001), but the median 6-month retention score of 74% (65–82%) was not significantly different than the post-course score (*p* = 0.65). Similarly, for the skills evaluation, the median post-course score of 86% (81–89%) was significantly higher than the pre-course score of 16% (7–45%) (*p* = 0.003) with no significant difference in the median 6-month retention score of 88% (88–92%) (*p* = 0.36).

Learner scores on the same evaluation tools were compared between curricula. There was no significant difference in performance in the post-course scores for written knowledge (*p* = 0.36) or skills (*p* = 0.95), nor in the 6-month retention scores for written knowledge (*p* = 0.28) or skills (*p* = 0.33). Additionally, neither group of learners came in with different levels of capability for the written knowledge (*p* = 0.49) or the skills (*p* = 0.18) as assessed by pre-course testing.

In terms of resource utilization, the Blended Learning Curriculum required 4 h and 50 min of faculty-hours during Step I, where faculty-hours are measured by number of instructors multiplied by hours required. In contrast, the Traditional Curriculum required 20 faculty-hours of instruction during Step I. This represents 15.16 faculty-hours saved.

Participants completed a course evaluation with questions answered using a Likert scale, where 1 meant “Strongly Disagree” while 5 meant “Strongly Agree” (Table 4). Course ratings were positive, with 100% of learners responding that they felt the course was well designed and engaging. On voluntarily provided open-ended text responses, learners reinforced the utility of the course and noted that the required asynchronous modules and readings were “manageable in volume.” Specifically, one learner noted “pre-readings helped give context” and that “SonoSim interactive cases using the probe were somewhat useful just to enforce some of the positioning and movements” but that “in-class didactics and ability to scan the models during the teaching was the most valuable” component of the course. Another participant appreciated “the organization was very structured” and the course content was “practical.” Constructive feedback emphasized a desire for more in-person scanning sessions and that more time could have been spent on cardiac echocardiography and other calculation-heavy topics.

**Table 4:** Course evaluation feedback from participating learners in blended learning curriculum. 9 (100%) PCCM fellows completed a course evaluation, where responses were provided on a Likert scale where 1 = strongly disagree and 5 = strongly agree

Prompt	Rating^a^
The course was well designed	5.00
I felt engaged for the majority of the course time	5.00
Testing was fair	4.88
There was enough time allotted for the course overall	4.88
Chosen topics were relevant to my practice	5.00
MICU scanning sessions were helpful	4.75
I would recommend the SonoSim curriculum for future PCCM Fellowship trainees in the coming years	4.88

^a^Assessed using a Likert scale (1 = Strongly disagree, 5 = Strongly agree)

## Discussion

Bedside US training remains variable amongst PCCM fellowships due to lack of an established curriculum. Maturation of the clinician-sonographer includes the ability to acquire accurate imaging and interpret the imaging within clinical context. A multi-faceted approach such as the curriculum outlined here is likely to be appropriate to meet this goal given its use of numerous methods of US training known to be effective independent of each other. Our Blended Learning Curriculum for US proved as effective as our prior traditional in-person course while saving faculty-hours. These findings are impactful given that faculty-time and expertise is a commonly cited barrier to standardized ultrasound education [2–4, 6, 8]. We saw a significant increase in US knowledge and skills among PCCM fellows who participated in the course which was retained after 6 months. The course was very well-received by learners with Likert scale-based ratings averaging 4.75-5 across the evaluation elements.

Importantly, switching our curriculum to omit in-person didactics led to comparable performance immediately post-course and 6 months post-course. This approach imparts benefits for flexibility of fellows, who may find it difficult to attend several in-person didactics interspersed between clinical responsibilities. Additionally, portfolio development during clinical responsibilities with intermittent review allows learners to continue their learning during clinical responsibilities, with review and consolidation of learning occurring at times most convenient for learners. Qualitative feedback offered by learners revealed that in-person didactics were still valuable and necessary for the execution of the curriculum. We posit that learners benefit from both the personalized attention of a traditional curriculum’s in-person didactics and hands-on time, and from the autonomy and flexibility of asynchronous didactics. The Blended Learning Curriculum provides both of these elements while mitigating the time and person-power barriers commonly faced by fellowship programs.

Our curriculum was organized by one ultrasound educator faculty for a fellowship program of 4–6 trainees per year with varying levels of prior training ranging from no experience to completion of a formal course. We believe a blended learning approach, which has been shown to save faculty-hours, is generalizable to fellowships of a similar size and with similar available faculty. Fellowships of a significantly smaller size may find a model with greater asynchronous didactics to be more suitable, while fellowships of a significantly larger size with an abundance of trained faculty may elect for more in-person didactics.

Limitations of our study include implementation at a single institution and small sample size. Our study is further limited by a lack of randomization which would allow a simultaneous comparison of the two approaches, rather than our comparison which is made across fellowship classes. A limitation of the blended learning approach is that fellowships must invest in a learning platform such as SonoSim which may be cost-prohibitive unless they elect to produce their own didactic materials.

Further, a potential barrier to wide implementation of our curriculum is that trained faculty are needed for successful implementation of our program, though a Blended Learning curriculum would require fewer faculty and/or fewer faculty hours. To be sustainable, there needs to be a core of faculty who are US instructors to ensure ongoing quality of trainee work. Currently, there are national training courses for practicing attending physicians who wish to serve this role for their institutions. Finally, it should be noted that while we have described our full educational paradigm, we have provided an evaluation of the Blended Learning Curriculum’s asynchronous format in Step 1 of the training paradigm as a potential for mitigating barriers to formal course-style education which is known to be prohibitive due to excess demand on faculty-hours and expertise. The data within our report cannot speak to further development of competency or benefits to faculty-hours achieved by later steps of the Blended Learning Curriculum although literature has described in isolation the efficacy of modalities employed by these steps [14, 15]. 

Future directions could include a formal randomized trial within or between fellowships on an approach incorporating blended didactics and a fellowship incorporating in-person didactics. Similarly, a standardized model for US education in PCCM fellowship has not yet been established and would allow better comparison of educational models on metrics of faculty-hours and trainee-hours to achieve identical learning objectives. We believe our study and associated supplemental material could inform the development of a standardized model which incorporates shared blended learning didactics or serve as a basis for development of more externally validated assessment tools.

## Conclusions

This work outlines a model for a longitudinal US training curriculum in PCCM fellowship. Comparison was made between a blended learning model, with asynchronous modules, and a traditional model, with in-person didactics. Both programs performed comparably and showed a significant improvement in both knowledge and skills immediately post-course and six months after the conclusion of Step I of the course, with the blended learning model saving 15 faculty-hours. This work additionally outlines the remainder of an ultrasound curriculum incorporating pedagogies including development of an image portfolio, clinically targeted sessions, training the trainer sessions and a summative OSCE.

## Electronic supplementary material

Below is the link to the electronic supplementary material.

Additional file 1: Step 1

Additional file 2: Step 2

Additional file 3: Step 3

Additional file 4: Step 4

Additional file 5: OSCE

Additional file 6: OSCE Videos

Additional file 7: OSCE Key

Additional file 8: Knowledge Assessment

## Data Availability

The dataset analyzed in this study is available from the corresponding author on reasonable request.

## Abbreviations

US: Bedside ultrasound
PCCM: Pulmonary and critical care medicine
IQR: Interquartile range
ACGME: Accreditation Council for Graduate Medical Education
CCM: Critical care medicine
ICU: Intensive care unit
OSCE: Objective structured clinical examination
