# Supplementary material for: A longitudinal blended learning curriculum for bedside ultrasound education in pulmonary and critical care fellowship
Source: BMC Med Educ. 2025 Jan 24;25:123. doi: 10.1186/s12909-024-06584-8 (PMC11762126; doi:10.1186/s12909-024-06584-8)
Supplement: Supplementary file 1 — Additional file 1: Step 1 [file 12909_2024_6584_MOESM1_ESM.docx]

**Program Step I, Formal Coursework**

**I. SonoSim Modules**

*This list of modules may be used in concert with the SonoSim platform, or as a reference for the list of topics and skills covered during this comprehensive ultrasound course.*

**Sessions 1-3: Introduction, Fundamentals, and Vascular Access and Diagnostics**

| Topic | SonoSim Module | SonoSim Screen Numbers | Time |
| --- | --- | --- | --- |
| Fundamentals of Ultrasound | Introduction | 2-4 | 00:03:27 |
| Fundamentals of Ultrasound | Basic Ultrasound Physics | 1-4 | 00:06:32 |
| Fundamentals of Ultrasound | Ultrasound System Operation | 9-11 | 00:03:26 |
| Fundamentals of Ultrasound | Basic Ultrasound Physics | 5 | 00:01:03 |
| Fundamentals of Ultrasound | Imaging Artifacts | 1-5; 13-15; 17-18 | 00:06:52 |
| Fundamentals of Ultrasound | Transducer Basics | 1-5 | 00:05:30 |
| Fundamentals of Ultrasound | Imaging Modes | 1-2 | 00:01:16 |
| Fundamentals of Ultrasound | Ultrasound System Operation | 3-4 | 00:01:27 |
| Fundamentals of Ultrasound | Ultrasound System Operation | 1-2 | 00:00:41 |
| Ultrasound-Guided Internal Jugular Vein Cannulation | Regional Anatomy | 1-3 | 00:03:34 |
| Ultrasound-Guided Internal Jugular Vein Cannulation | Regional Anatomy | 4 | 00:00:40 |
| Ultrasound-Guided Internal Jugular Vein Cannulation | Sonographic Anatomy | 1-4 | 00:03:43 |
| Ultrasound-Guided Internal Jugular Vein Cannulation | Imaging Adjuncts | 2-4 | 00:01:54 |
| Ultrasound-Guided Internal Jugular Vein Cannulation | Procedure Preparation  Procedure Steps | 1-4, 7-11  1-4, 7-12, 15-17 | 00:25:20 |
| Ultrasound-Guided Femoral Line Placement | Regional Anatomy  Sonographic Anatomy  Procedure Preparation  Procedure Steps | 1-2, 6-8  1-10  5-6  1-13 | 00:05:32  00:12:52  00:01:53 |
| Subclavian Vein | Regional Anatomy  Sonographic Anatomy  Procedure Steps | 1-3  1-8  1-7, 10-13 | 00:04:05  00:06:34  00:11:41 |
| Arm-Arterial: Anatomy & Physiology | Sonographic Anatomy  Sonographic Technique | 1-6  1-5, 10-11, 13 | 00:00:44  00:05:31 |
| Leg-Venous: Anatomy & Physiology | Sonographic Anatomy | 1-8 | 00:04:54 |
| Leg-Venous: Anatomy & Physiology | Sonographic Technique | 1-2 | 00:01:33 |
| Leg-Venous: Anatomy & Physiology | Sonographic Technique | 10-11 | 00:01:08 |
| Rapid Ultrasound in Shock (RUSH) | The Pipes: DVT | 1-8 | 00:03:20 |
| Leg-Venous: Anatomy & Physiology | Anatomy  Sonographic Technique | 1-3  3-9 | 00:03:11  00:07:04 |
| Total Module Time | | | **02:28:45** |
| Total In-Person Didactic Time (including scanning and imaging review) | | | **00:30:00** |

***Scanning Objectives***

*These objectives must be achieved either by virtual simulation (e.g. SonoSim platform) or in-person practice.*

| **Objective** | **SonoSim Training Module** | **Case #** |
| --- | --- | --- |
| Internal Jugular Access | Procedures 🡪 Internal Jugular Vein Access | 1-10 |
| Carotid Artery | Vascular 🡪 Basic Cerebrovascular | 1-3 |
| DVT Study | Protocols 🡪 RUSH | 8 (right and left groin) |
| Femoral Line Placement | Procedures 🡪 Femoral Vein Access | 1-10 |
| Subclavian Vein | Procedures 🡪 Subclavian Vein Access | 1-10 |
| Arterial Arm | Vascular 🡪 Basic Arm-Arterial | 1-3 |
| Leg-Venous | Vascular 🡪 basic Leg- Venous | 1-3 |
| Tip-tracking/Transducer direction | In-person didactic: 20 minutes | |

***Imaging Review***

*These topics were selected for further review due to clinical importance.*

-Review DVT studies; not very thoroughly covered with SonoSim Course or Interactive Modules (10 minutes)

**Session 4: Pulmonary Ultrasound**

| **Topic** | **SonoSim Module** | **SonoSim Screen Numbers** | **Time** |
| --- | --- | --- | --- |
| Lungs: Anatomy & Physiology | Sonographic Technique | 1; 4-5 | 00:02:09 |
| Lungs: Anatomy & Physiology | Sonographic Anatomy | 1-3 | 00:01:52 |
| Pulmonary | Lung Evaluation | 4 | 00:01:11 |
| Lungs: Anatomy & Physiology | Sonographic Anatomy | 6-9 | 00:02:27 |
| Pulmonary | Pneumothorax Rule-Out | 3-6 | 00:03:24 |
| N/A | Chest Tube | In-person didactic: 5 minutes | |
| Lungs: Anatomy & Physiology | Sonographic Technique | 6 | 00:01:06 |
| Pulmonary | Pneumothorax Rule-In | 1-3; 5-6, 8-9 | 00:05:46 |
| Pulmonary | Imaging Adjuncts | 2-3 | 00:01:46 |
| Pulmonary | Pneumothorax Rule – Out | 1 | 00:00:36 |
| Lungs: Anatomy & Physiology  Pulmonary  Pulmonary | Sonographic Anatomy  Imaging Adjuncts  Imaging Artifacts | 10-11  7  1-2 | 00:01:14 |
| N/A | Pulmonary Edema | In-person didactic: 10 minutes | |
| Pulmonary | Lung Consolidation | 1-2, 4-8 | 00:04:05 |
| N/A | Thoracentesis | In-person didactic: 10 minutes | |
| Pulmonary | Pleural Effusion | 1-6 | 00:02:05 |
| N/A | Exudate/Transudate; Septations/Loculations | In-person didactic: 10 minutes | |
| Airway | Hemidiaphragm Sonography | 1-8 | 00:03:29 |
| Airway | Imaging Basics  Imaging  Adjuncts  Endotracheal Tube Localization  Endotracheal Intubation Confirmation | 1-7  4-5, 13-14  1-12  1-8 | 00:02:48  00:03:37  00:04:53  00:04:34 |
| **Total Module Time** | | | **00:47:02** |
| **Total In-Person Didactic Time (including scanning and imaging review)** | | | **01:25:00** |

***Scanning Objectives***

| **Objective** | **SonoSim Training Module** | **Case #** |
| --- | --- | --- |
| Basic Lung | Pulmonary 🡪 Basic Lungs | 1-3 |
| Anterior Chest Wall (lung sliding) | Pulmonary 🡪 Core Pulmonary | 1-4 |
| Eval. Of Pneumothorax | Pulmonary 🡪 Core Pulmonary | 5 |
| Eval. Of Pulmonary Edema/Pleural Effusion | Pulmonary 🡪 Core Pulmonary | 6-10 |
| Eval. Of Airway | Head/Neck 🡪 Core Airway | 1-5 |
| Thoracentesis | In-person didactic: 5 minutes | |
| Effusions | In-person didactic: 5 minutes | |

***Imaging Review***

- Grading Pulmonary Edema (20 minutes)
- Effusions including exudative/transudative, septations vs loculations (20 minutes)

**Sessions 5-6: Abdominal, Extremity Ultrasound and IVC**

| **Topic** | **SonoSim Module** | **SonoSim Screen Numbers** | **Time** |
| --- | --- | --- | --- |
| FAST Protocol | Background | 8-10 | 00:01:12 |
| FAST Protocol | RUQ Views  LUQ Views | 6-9  1-3 | 00:01:15  00:01:45 |
| FAST Protocol | Pelvic Views | 1-6 | 00:02:24 |
| FAST Protocol | Cardiac Views | 1-2 | 00:01:29 |
| Renal | Sonographic Anatomy | 2-3 | 00:01:06 |
| Renal | Sonographic Anatomy | 1 | 00:00:43 |
| Renal | Obstructive Uropathy | 3-5 | 00:02:52 |
| Aorta/IVC | Aortic Anatomy  Imaging Technique | 1-3; 5-9  1-4 | 00:03:47  00:03:24 |
| N/A | Paracentesis | In-person didactic: 5 minutes | |
| N/A | Evaluation of Pneumoperitoneum | In-person didactic: 20 minutes | |
| GI Tract: Anatomy & Physiology | Anatomy | 9 | 00:01:35 |
| GI Tract: Anatomy & Physiology | Sonographic Anatomy | 13 | 00:00:25 |
| Aorta/IVC  Rapid Ultrasound in Shock (RUSH) | IVC Evaluation and CVP  IVC Case Studies  The Tank: Intravascular volume | 1-5  1-2  1-9 | 00:03:24  00:00:42  00:02:35 |
| N/A | IVC Distensibility Index | In-person didactic: 25 minutes | |
| Arm-Arterial: Anatomy & Physiology | Sonographic Anatomy  Sonographic Technique | 1-6  1-5, 12 | 00:00:44  00:04:35 |
| **Total Module Time** | | | **00:33:57** |
| **Total In-Person Didactic Time (including scanning and imaging review)** | | | **01:05:00** |

***Scanning Objectives***

| **Objective** | **SonoSim Training Module** | **Case #** |
| --- | --- | --- |
| FAST Protocol | Protocols 🡪eFAST | 1-10 |
| Renal Views | Abdomen 🡪 Basic Renal  Core Renal | 1-3  1-10 |
| Aorta/IVC | Vascular 🡪Basic Aorta/IVC | 1-3 |
| GI Tract | Abdomen 🡪 Basic GI Tract | 1-3 |
| RUSH | Protocols 🡪RUSH | 1-10 |
| Paracentesis | In-person didactic: 5 minutes | |
| Eval of Pneumoperitoneum | In-person didactic: 5 minutes | |

***Image Review***

- Pneumoperitoneum (5 minutes)

**Sessions 7-8: Echocardiography**

| **Topic** | **SonoSim Module** | **SonoSim Screen Numbers** | **Time** |
| --- | --- | --- | --- |
| Cardiology | Introduction  Imaging Conventions | 1-11  1-5 | 00:05:31  00:06:07 |
| Cardiology | Parasternal Long-Axis View  Parasternal Short-Axis View: Mid-Ventricle  Parasternal Short Axis View: Base | 1-8  1-6  1-6 | 00:04:46  00:03:12  00:03:46 |
| Cardiology | Apical 4 Chamber View | 1-6 | 00:04:17 |
| Cardiology | Subcostal Four-Chamber View | 1-7 | 00:04:07 |
| Cardiology | IVC Evaluation & RAP | 1-10 | 00:05:43 |
| N/A | LV Dysfunction | In-person didactic: 20 minutes | |
| N/A | IVC and RV Filling | In-person didactic: 5 minutes | |
| N/A | Vegetations | In-person didactic: 15 minutes | |
| N/A | Infarcted Mitral Valve | In-person didactic: 15 minutes | |
| Cardiology | Case Studies | 6-11 | 00:02:27 |
| N/A | Hypotension, Shock States | In-person didactic: 20 minutes | |
| Cardiology | Case Studies | 1-5 | 00:02:20 |
| N/A | Additional Case Studies* | In-person didactic: 15 minutes | |
| **Total Module Time** | | | **00:40:14** |
| **Total In-Person Didactic Time (including scanning and imaging review)** | | | **01:30:00** |

***Scanning Objectives***

| **Objective** | **SonoSim Training Module** | **Case #** |
| --- | --- | --- |
| Echo | Cardiac🡪Basic Heart | 1-3 |
| Cardiac Cases | Cardiac 🡪 Core Cardiology & Adv. FoCUS | 1-20 & 1-10 |

* Cardiac cases include the following concepts: pericardial effusion w/swinging heart sign, mitral annular calcification, decreased EF, aortic valve calcification, LVH, end-diastolic right ventricular collapse, dilated cardiomyopathy, pulmonic and tricuspid regurgitation, hyperdynamic heart, aortic and mitral regurgitation, aortic dissection, L atrial mass, decreased biventricular contractility, pulmonary hypertension

*Note that the Blended Learning curriculum had a higher number of echo cases than the Traditional Curriculum due to a higher number of cases in the online SonoSim repository compared to what faculty could gather alone.*

**Session 9: Critical Care Ultrasound**

| **Topic** | **SonoSim Module** | **SonoSim Screen Numbers** | **Time** |
| --- | --- | --- | --- |
| Rapid Ultrasound in Shock (RUSH) | Introduction  The Pump Global LV Function | 1-9  1-14 | 00:05:31  00:06:07 |
| N/A | Modified BLUE Protocol | In-person didactic: 5 minutes | |
| N/A | Reverse-FALLS Protocol | In-person didactic: 5 minutes | |
| FAST Protocol | Imaging Artifacts & Pitfalls | 5-7 | 00:01:12 |
| N/A | Cardiac Arrest Protocol | In-person didactic: 10 minutes | |
| **Total Module Time** | | | **00:12:50** |
| **Total In-Person Didactic Time** | | | **00:20:00** |

**II. Additional Self-Directed Learning Resources***Additional resources including primary literature are provided for review prior to and during the year-long curriculum.*

1. Online resources:
   1. http://www.susme.org/learning-modules/learning-modules/
      1. Introduction
      2. Neck anatomy, femoral vessel anatomy
      3. Lung ultrasound
      4. Abdominal ultrasound and the Focused Assessment with Sonography for Trauma (FAST) exam.
      5. Echocardiography
2. Textbook Chapters:
   1. Mayo PH. Critical care echocardiography. In: Bolliger CI, Herth FJF, Mayo PH, Miyazawa T, Beamis JF eds. Clinical Chest Ultrasound: From the ICU to the Bronchoscopy Suite. Karger, 2009: vol 37, pp 60-68.
   2. Ahmad S, Eisen LA. Lung ultrasound: The basics. In: Lumb P, Karakitsos D eds. Critical Care Ultrasound; Elsevier; 2014. Chp 19, pp 106-110.
3. Literature List:
   1. Moore CL and Copel JA. Point-of-care ultrasonography. N Engl J Med 2011;364:749-57.
   2. Mayo PH, Beaulieu Y, Doelken P, et al. American college of chest physicians/ la societe de reanimation de langue francaise statement on competence in critical care ultrasonography. CHEST 2009;135;1050-60.
   3. Kory PD, Pellecchia CM, Shiloh AL, et al. Accuracy of ultrasonography performed by critical care physicians for the diagnosis of dVT. CHEST 2011; 139(3):538–42.
   4. DiBello C and Koenig S. Diagnosis of deep venous thrombosis by critical care physicians using compression ultrasonography. The Open Critical Care Medicine Journal 2010; 3:43-7.
   5. Chen SC, Yen ZS, Wang HP et al. Ultrasonography is superior to plain radiography in the diagnosis of penumoperitoneum. Brit J Surg 2002;89: 351-4.
   6. Hoffmann B, Nurnberg D and Westergaard C. Focus on abnormal air: diagnostic ultrasonography for the acute abdomen. Eur J Emerg Med 2012; 00(00):1-8.
   7. Lichtenstein DA, Meziere GA, Lagouyete J-F, et al. A-Lines and B-Lines. Lung ultrasound as a bedside tool for predicting pulmonary artery occlusion pressure in the critically ill. CHEST 2009; 136(4):1014-20.
   8. Noble VE, Murray AF, Capp R, et al. Ultrasound assessment for extravascular lung water in patients undergoing hemodialysis. CHEST 2009; 135:1433–9.
   9. Alrajhi K, Woo MY and Vaillancourt C. Test characteristics of ultrasonography for the detection of pneumothorax. A systematic review and meta-analysis. CHEST 2012; 141(3):703–8.
   10. Zanforlin A, Gavelli G, Oboldi D, et al. Ultrasound-guided thoracenthesis: the V- point as a site for optimal drainage positioning. Eur Rev Med Pharmaco 2013. 17:25-8.
   11. McCool FD and Tzelepis GE. Dysfunction of the diaphragm. N Engl J Med 2012. 366;(10):932-42.
   12. Barbier C, Loubieres Y, Schmit C, et al. Respiratory changes in inferior vena cava diameter are helpful in predicting fluid responsiveness in ventilated septic patients. Intens Care Med 2004; 30:1740–6.
   13. Yanagawa Y, Sakamoto T and Okada Y. Hypovolemic shock evaluated by sonographic measurement of the inferior vena cava during resuscitation in trauma patients. J Trauma 2007; 63(6):1245-8.
   14. Muller L, Bobbia X, Toumi M, et al. Respiratory variations of inferior vena cava diameter to predict fluid responsiveness in spontaneously breathing patients with acute circulatory failure: need for a cautious use. Crit Care 2012; 16:R188.
   15. Fremont B, Pacouret G, Jacobi D, et al. Prognostic value of echocardiographic right/left ventricular end-diastolic diameter ratio in patients with acute pulmonary embolism. CHEST 2008; 133:358–362.
