# Supplementary material for: A longitudinal blended learning curriculum for bedside ultrasound education in pulmonary and critical care fellowship
Source: BMC Med Educ. 2025 Jan 24;25:123. doi: 10.1186/s12909-024-06584-8 (PMC11762126; doi:10.1186/s12909-024-06584-8)
Supplement: Supplementary file 2 — Additional file 2: Step 2 [file 12909_2024_6584_MOESM2_ESM.docx]

**Program Step II, Image Portfolio Development by Level**

| Level | Images |
| --- | --- |
| **Level I**  1 month post-course  *Basic normal findings* | Lung sliding in M-mode |
|  | A-lines |
|  | Hepatorenal recess (include kidney, liver and diaphragm) |
|  | Saphenofemoral junction |
|  | Common femoral vein and femoral artery proximal to saphenofemoral junction |
|  | Superficial femoral vein |
|  | Urinary bladder |
|  | PSLA^a^ view |
|  | PSSA^b^ view, at the level of the papillary muscles (mid-ventricular view) |
|  | Cardiac apical four chamber view |
|  | Cardiac subcostal four chamber view |
|  | IVC^c^, longitudinal view, in M-mode |
|  | Aorta, longitudinal view, B-mode in still frame |
| **Level II**  3 months post-course  *Identification of pathology and advanced abdomen* | B-lines |
|  | Pleural effusion, from midaxillary view |
|  | Bladder with Foley in place |
|  | Aorta |
|  | Long axis view at the superior mesenteric artery branch point |
|  | Long axis view at the iliac artery bifurcation |
|  | Short axis view with the IVC and splenic vein identified |
|  | Aorta measured in a short axis view (B-mode still frame) |
|  | Ascites |
|  | Gallbladder with measurement of anterior wall in B-mode still frame |
|  | Left kidney |
| **Level III**  6 months post-course  *Case development: one case covered as a case presentation* | Ultrasound approach to Cardiogenic shock |
|  | Ultrasound approach to Hypovolemic shock |
|  | Ultrasound approach to Septic shock |
|  | Ultrasound approach to Respiratory failure |
|  | Ultrasound approach to Fluid resuscitation |
|  | Ultrasound approach to Diuresis |
|  | Ultrasound approach to Renal failure |
|  | Ultrasound approach to Cardiac Arrest management |
| **Level IV**  1 year and beyond post-course  *Advanced bedside echo and pulmonary ultrasound* | LV functional assessment by M- Mode |
|  | Stroke Volume assessment by Velocity time integral (VTI) |
|  | RV inflow and outflow views |
|  | RV functional assessment by Tricuspid Annular Plane Systolic Excursion |
|  | Subcostal short axis view |
|  | Diaphragm thickening (right midaxillary line at the zone of apposition) |
| ^a^Parasternal long axis, ^b^Parasternal short axis, ^c^Inferior Vena Cava | |
