# Supplementary material for: A longitudinal blended learning curriculum for bedside ultrasound education in pulmonary and critical care fellowship
Source: BMC Med Educ. 2025 Jan 24;25:123. doi: 10.1186/s12909-024-06584-8 (PMC11762126; doi:10.1186/s12909-024-06584-8)
Supplement: Supplementary file 3 — Additional file 3: Step 3 [file 12909_2024_6584_MOESM3_ESM.docx]

**Program Step III, Deliberate Practice Plan**

Self-Perception

Self-Perceived Strengths:

Self-Perceived Deficiencies:

Image Portfolio Review

*Images from Level I and Level II of the fellow’s image portfolio are reviewed and critiqued at this time.*

Skills Assessment

“You will be asked to perform different tasks; perform each ultrasound examination as if on a real patient. This includes positioning yourself, the machine and the patient; if certain positioning or other movements are infeasible due to space or safety constrains, then simply verbalize your intentions; remember to optimize the image; when you achieve an image which you deem acceptable for the task at hand alert me and name the anatomy which is in view”

“Identify a safe site for IJ access”

| Positioning | Patient positioned supine or in trendelenberg |  |
| --- | --- | --- |
|  | Operator positioned at head of bed |  |
|  | Machine positioned in line of vision at arm’s length |  |
|  |  |  |
| Transducer and machine | High frequency linear array transducer in use |  |
|  | Transducer marker to operator left (or match screen) |  |
|  | Transducer held in cross section |  |
|  | Depth appropriately set |  |
|  | Gain appropriately set |  |
|  |  |  |
| Anatomy | Identifies IJ vein |  |
|  | Identifies carotid artery |  |
|  | Identifies the SCM muscle |  |
|  |  |  |
| Evaluation and technique | Compression of IJ to establish patency |  |
|  | Examination of IJ from jawline to base of neck |  |
|  | Selection of a mid- neck site |  |
|  | Orientation of vein and artery |  |
|  | “show with your finger the angle of your needle” |  |
|  |  |  |
| Completion of Evaluation | Assessment at contralateral IJ |  |
|  | Assessment for lung sliding at least ipsilaterally |  |
|  |  |  |
|  | Overall acceptable study |  |
|  | Total marks (out of possible 18) |  |

“Perform a DVT study on one leg using only 2D ultrasound; you may choose either leg or change sides even after you have started. State the anatomy as you go”

| Positioning | Patient low extremity externally rotated |  |
| --- | --- | --- |
|  | Operator positioned at side of bed |  |
|  | Machine positioned in line of vision at arm’s length |  |
|  |  |  |
| Transducer and machine | High frequency linear array transducer in use |  |
|  | Transducer marker to operator left (or match screen) |  |
|  | Transducer held in cross section |  |
|  | Depth appropriately set and adjusted during majority of study |  |
|  | Gain appropriately set and adjusted during majority of study |  |
|  |  |  |
| Anatomy | Identifies CFV proximal to SFJ |  |
|  | Identifies SFJ |  |
|  | Identifies bifurcation of femoral artery |  |
|  | Identifies CFV distal to the SFJ |  |
|  | Identifies deep femoral vein; or verbalizes expected location |  |
|  | Identifies popliteal vein |  |
|  |  |  |
| Evaluation and technique | Compression at each of above points |  |
|  | 90 degree compression technique |  |
|  | Obliteration of vessel lumen with juxtaposition of walls |  |
|  |  |  |
|  | Overall acceptable study |  |
|  | Total marks (out of possible 17) |  |

“Prepare to perform chest ultrasound” “Identify lung sliding in B mode then M mode” “Identify A-lines”

“Identify the right hemidiaphragm”

| Positioning | Patient positioned supine with exposed anterior thorax |  |
| --- | --- | --- |
|  | Operator positioned at side of bed |  |
|  | Machine positioned in line of vision at arm’s length |  |
|  |  |  |
| Transducer and machine | Low frequency phased array transducer prepared for use |  |
|  | Transducer marker cephalad |  |
|  | Machine set for abdominal setting |  |
|  | Depth appropriately set and adjusted during majority of study |  |
|  | Gain appropriately set and adjusted during majority of study |  |
|  |  |  |
| Anatomy | Identifies lung sliding in B-mode |  |
|  | Identifies lung sliding in M-Mode |  |
|  | Identifies A-lines |  |
|  | Identifies Right hemidiaphragm |  |
|  |  |  |
| Evaluation and technique | Decreases gain to emphasize the pleural line |  |
|  |  |  |
|  | Overall acceptable study |  |
|  | Total marks (out of possible 13) |  |

“Prepare to perform abdominal ultrasound”

“Identify the Right kidney in long axis then in short axis” “Locate the inferior pole of this kidney”

“Perform a FAST exam”

| Positioning | Patient positioned supine with exposed anterior thorax |  |
| --- | --- | --- |
|  | Operator positioned at side of bed |  |
|  | Machine positioned in line of vision at arm’s length |  |
|  |  |  |
| Transducer and machine | Low frequency phased array transducer prepared for use |  |
|  | Transducer marker cephalad |  |
|  | Machine set for abdominal setting |  |
|  | Depth appropriately set and adjusted during majority of study |  |
|  | Gain appropriately set and adjusted during majority of study |  |
|  |  |  |
| Anatomy | Identifies kidney in long axis |  |
|  | Identifies kidney in short axis |  |
|  | Identifies inferior pole of kidney in short axis |  |
|  | Identified bladder |  |
|  | Identifies hepato- renal recess |  |
|  | Identifies spleno- renal recess |  |
|  | Appropriately positions for right paracolic gutter |  |
|  | Appropriately positions for left paracolic gutter |  |
|  | Attempts to perform subcostal cardiac view |  |
|  |  |  |
|  | Overall acceptable study |  |
|  | Total marks (out of possible 17) |  |

“Prepare for bedside echocardiography” “Show me a PSLA view”

“Show me a short axis view where you would assess LV function and regional wall motion abnormality” “Show me an apical four chamber view”

“Show me a subcostal view and identify the anatomy”

“Show me the IVC in longitudinal view; Identify relevant anatomy”

“Take measurements as you would in a shock patient” “interpret these measurements if the patient was intubated. Not- intubated?”

| Positioning | Patient positioned supine with exposed anterior thorax |  |
| --- | --- | --- |
|  | Operator positioned at side of bed |  |
|  | Machine positioned in line of vision at arm’s length |  |
|  |  |  |
| Transducer and machine | Low frequency phased array transducer prepared for use |  |
|  | Machine set for cardiac setting and dot at operator right |  |
|  | Depth appropriately set and adjusted during majority of study |  |
|  | Gain appropriately set and adjusted during majority of study |  |
|  | Displays working knowledge of caliper functionality |  |
|  |  |  |
| Anatomy | Accomplishes adequate PSLA view |  |
|  | Identifies LV, LA, LVOT, RVOT |  |
|  | Identifies Ao valve, mitral valve |  |
|  | Identifies Descending Aorta |  |
|  | Identifies pericardium |  |
|  |  |  |
|  | Achieves adequate PSSA view at the level of the papillary muscles |  |
|  | Identifies regions: septal, anterior, lateral, inferior |  |
|  | Identifies RV |  |
|  |  |  |
|  | Achieves adequate A4C view |  |
|  | Identifies LV, LA, RV, RA, Mitral valve, Tricuspid valve |  |
|  |  |  |
|  | Achieves adequate Subcostal 4 chamber view |  |
|  | Identifies LV, LA, RV, RA, Mitral valve, Tricuspid valve |  |
|  |  |  |
|  | Achieves adequate view of IVC in longitudinal section at mid-  abdomen |  |
|  | Identifies cavo-atrial junction |  |
|  | Identifies Aorta in longitudinal section |  |
|  | Measures at 2-4cm from cavo-atrial junction |  |
|  |  |  |
| Knowledge | Interprets IVC measurements appropriately for PPV |  |
|  | Interprets IVC measurements appropriately for non-PPV |  |

|  | “How would you maneuver the transducer to move from PSSA  view at level of papillary muscle to level of aortic valve” |  |
| --- | --- | --- |
|  | “Which cardiac view would you use to evaluate RV function and  size” |  |
|  | “What finding on PSSA would suggest RV volume or pressure  overload? |  |
|  | “What is one alternative way to view the IVC if anterior- abdomen  is unavailable” |  |
|  |  |  |
|  | Overall acceptable study |  |
|  | Total marks (out of possible 30) |  |

“Start a new US session on the sonosite; label it with your initials; show me the abdominal aorta in cross section” “Save a still image of the abdominal aorta”

“Show me peristalsis; save a 6 second prospective clip of peristalsis” “Prepare the machine to upload your images to a USB drive”

| Machine control | Displays working knowledge of still image save functionality |  |
| --- | --- | --- |
|  | Displays working knowledge of clip functionality |  |
|  | Displays working knowledge of upload functionality |  |
|  |  |  |
| Anatomy | Identifies Aorta in short axis |  |
|  | Identifies gut sliding |  |
|  | Identifies peristalsis |  |
|  |  |  |
|  | Overall acceptable study |  |
|  | Total marks (out of possible 6) |  |

Fellow:

Instructor:
