# Supplementary material for: A longitudinal blended learning curriculum for bedside ultrasound education in pulmonary and critical care fellowship
Source: BMC Med Educ. 2025 Jan 24;25:123. doi: 10.1186/s12909-024-06584-8 (PMC11762126; doi:10.1186/s12909-024-06584-8)
Supplement: Supplementary file 4 — Additional file 4: Step 4 [file 12909_2024_6584_MOESM4_ESM.docx]

**Program Step VI, Training the Trainer**

*The following instructions were provided to trainers prior to leading a teaching session.*

**Room Configuration and Teaching Guidelines**

Standardized Patient (SP), i.e., the model, should already have shorts and gown open to the front.
Ask the SP to tuck one towel into front of shorts and one at the shorts’ leg:


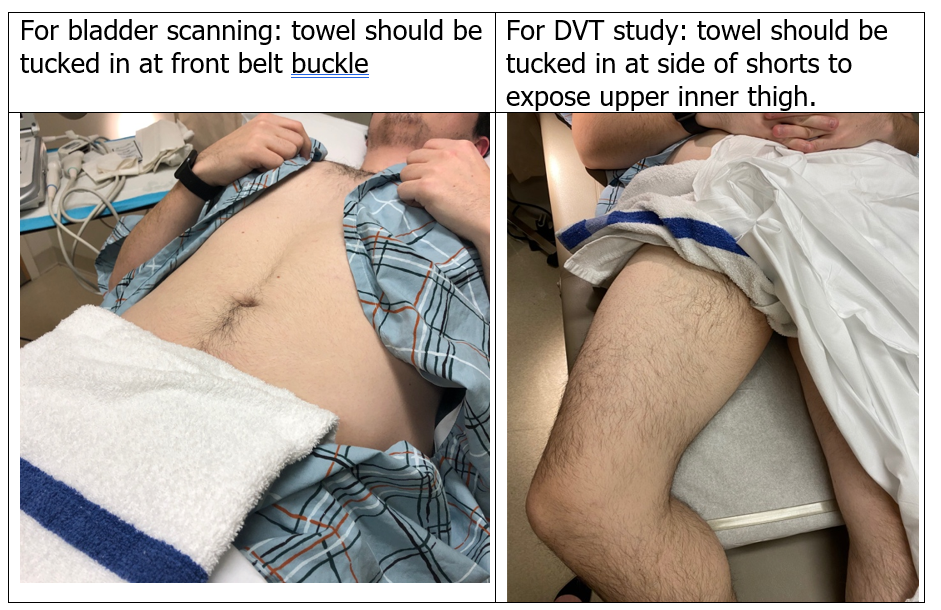


Set up the ultrasound machine at the SP's right shoulder/head. Ensure the machine is plugged in. At the start of the session have the Linear Array High Frequency probe connected and engaged. Use "Venous" setting.

You will stand on the opposite side of the learner. Reaching across as needed and co-hold the probe if the learner needs help. All the while, you should be verbalizing to the learner what adjustments they should make on the machine.

Remember to ask "I’ll hold with you if you don’t mind. Is that okay?"


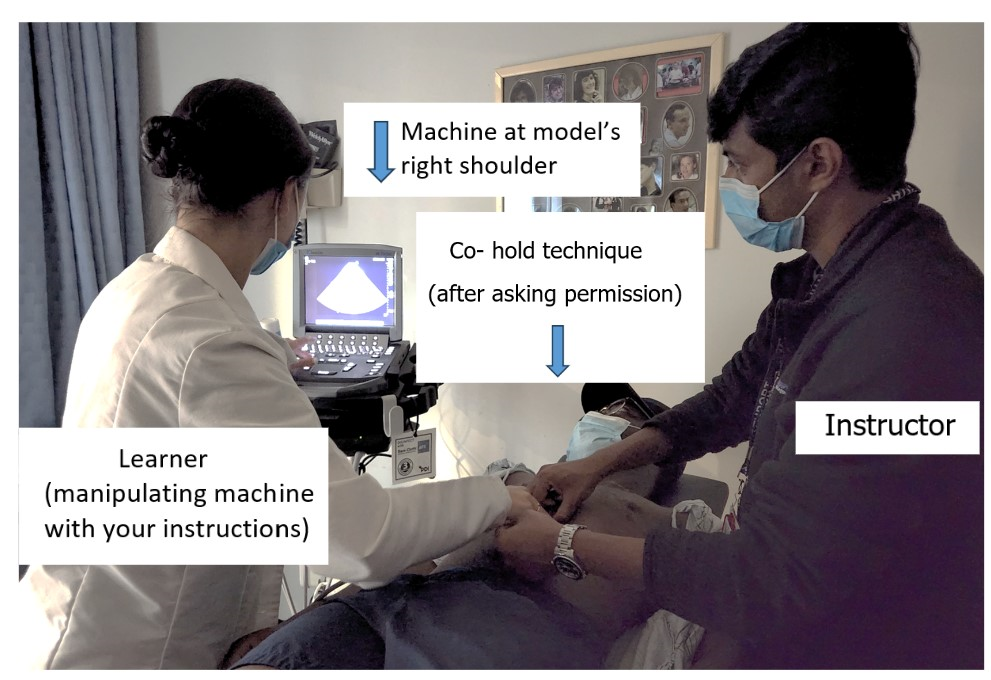


Ensure hands-on time for each of the students for each of the sessions.

This is hard to do. It requires for you to force yourself not to hold or grab the probe, and to limit your own speaking even when you think you are helping by answering a question.

No demonstrations. Do not demonstrate the ultrasound technique yourself. Simply state to one of the learners, "Why don't you get us started? I'll help" and "If you don’t mind, I will hold the probe with you a bit." As the learner gets started, from across the opposite side of the SP, you reach over and help them handle the probe. You ask them to make the changes in the machine. The learner must do all of the hands on and machine changes. They can do it, you just have to:

1) be a little patient

2) if time is wasting by say "let’s come back to this" and move on

3) verbalize the instructions for what they must change on the machine

4) accept imperfect images.

**Learning Objectives**(didactics in gray, hands-on objectives are in blue):

*These sessions were conducted alongside lecture-based didactics; these can be substituted by prior preparation by learners or omission of these topics depending on the level of experience of the learners.*

| **Introduction to US**  **Physics**  **Probes**  **Ultrasound Modes**  **Scan Planes**  **Depth & Gain**  **Utility and scope of bedside US** |
| --- |
| **Vascular US**  **Vascular Access: pIV**  **Vascular Access: CVC**  **DVT Study** |
| **Introduction**  **Switch between probes**  **Gain, depth**  **IJ anatomy and compression technique**  **Peripheral IV placement (rotating blue phantom task trainers)** |
| **Chest Ultrasound**  **Normal chest**  **Pneumothorax**  **Pulmonary edema**  **Pleural effusion** |
| **Anterior Chest**  **Lung Sliding: High Frequency, B mode**  **Lung Sliding: High Frequency, M mode**  **Lung Sliding: Low Frequency, B mode**  **A-lines: Low Frequency B mode**  **Posterior-Lateral Chest**  **Right: Identify Kidney, Liver, Diaphragm, Lung artifact (Curtain Sign)** |
| **Abdomen**  **FAST Exam** |
| **FAST exam (except Subcostal heart view)**  **RUQ view**  **LUQ view**  **R paracolic gutter**  **L paracolic gutter**  **Bladder (transverse section)** |
| **Bedside Echocardiography & IVC**  **Scope and practice**  **Subcostal 4 chamber view**  **IVC** |
| **PSLA view**  **Subcostal 4 chamber view**  **IVC**  **Anterior subcostal longitudinal view:**  **B mode, M mode** |
