# Supplementary material for: A longitudinal blended learning curriculum for bedside ultrasound education in pulmonary and critical care fellowship
Source: BMC Med Educ. 2025 Jan 24;25:123. doi: 10.1186/s12909-024-06584-8 (PMC11762126; doi:10.1186/s12909-024-06584-8)
Supplement: Supplementary file 5 — Additional file 5: OSCE [file 12909_2024_6584_MOESM5_ESM.docx]

**Ultrasound OSCE**

*Answer sheet to complete is included at the end of the document.*

**Case Description**

**CC:** Respiratory distress, Shock. Rapid response team (RRT) called.

**HPI:** 54 year old male w/ HCV (untreated) c/b cirrhosis, alcohol abuse, h/o IVDU, R renal cell carcinoma s/p nephrectomy, presented with abdominal distension and decompensated cirrhosis.

This admission, patient had paracentesis (removed 6.7 L), negative for SBP. He has had 9 paracenteses in the last three months. He underwent TIPS for refractory ascites the day before the RRT was called. Noted to have drop in Hgb as well.

RRT was called for worsening mental status, respiratory distress.

**PMHx:** HCV, cirrhosis, alcohol abuse, h/o IVDU, R RCC s/p nephrectomy, Factor XI def

**PSHx:** R nephrectomy

**FHx:** Breast cancer, Uterine cancer, Lung cancer

**Social Hx:** Alcohol use [mostly beer], +cocaine and marijuana, +used to be a taxi drive, +smoker

**ROS:** unable to obtained

**Exam:**

**Vital Signs (last 24 hrs)_____ Last Charted___________**

**Temp Axillary** 35.9 DegC (APR 05 10:26)

**Resp Rate** 28 br/min (APR 05 11:46)

**SBP** 90 mmHG (APR 05 11:46)

**DBP** 68 mmHG (APR 05 11:46)

**SpO2** **L** 90% (APR 05 11:46) on room air

**Weight** 82 kg (APR 05 07:12)

**General**: Moderate respiratory distress.

**Eye**: Pupils are equal, round and reactive to light.

**HENT**: Normocephalic, Dry oral mucosa, +jaundice.

**Neck**: Supple.

**Respiratory**: Lungs are clear to auscultation.

**Cardiovascular**: Tachycardia

**Gastrointestinal**: Distended, tenderness at flanks.

**Genitourinary**: No costovertebral angle tenderness.

**Lymphatics**: No lymphadenopathy neck, axilla, groin.

**Integumentary**: Warm.

**Musculoskeletal**: Normal range of motion.

**Labs:**

|  | **pH - ART** | **7.28 LOW** | | |
| --- | --- | --- | --- | --- |
|  | **PCO2 - ART** | **30 mmHg LOW** | | |
|  | **PO2 - ART** | **73 mmHg LOW** | | |
|  | **HCO3 - ART** | **14 mEq/L LOW** | | |
|  | **BD (Art)** | **13.2 mEq/L HI** | | |
|  | O2 Hb - ART | 95 % | | |
| Sodium | | | 136 mmol/L |  |
| **Potassium** | | | **6.2 mmol/L** |  |
| Chloride | | | 107 mmol/L |  |
| **Bicarbonate** | | | **16 mmol/L LOW** |  |
| Glucose Level | | | 74 mg/dL |  |
| **BUN** | | | **48 mg/dL HI** |  |
| **Creatinine** | | | **1.77 mg/dL HI** |  |
| **Bilirubin, Total** | | | **4.2 mg/dL HI** |  |
| **Bilirubin, Direct** | | | **2.4 mg/dL HI** |  |
| **ALT (SGPT)** | | | **91 IU/L HI** |  |
| **AST (SGOT)** | | | **201 IU/L HI** |  |
| **Alk Phos** | | | **93 IU/L** |  |
| **Albumin** | | | **3.2 g/dL LOW** |  |
| **Total Protein** | | | **4.9 g/dL LOW** |  |
| **WBC Count** | | | **In Lab K/uL** |  |
| **RBC Count** | | | **1.73 M/uL LOW** |  |
| **Hemoglobin** | | | **6.1 g/dL** |  |
| **Hematocrit** | | | **17.5 %** |  |
| **MCV** | | | **101.2 fL HI** |  |
| **PLT Count** | | | **63 K/uL** |  |
|  | | |  |  |
|  | | |  |  |

**Imaging:** *Please see separate PowerPoint file (Appendix F) for ultrasound imaging.*

**Answer Sheet**

**Exam:** Limited chest, Limited ECHO, Limited abdomen

**Indication:** Shock state, Respiratory distress

**Findings:**

Lung US:

ECHO:

Abdomen:

Interpretation:

What would be your immediate management for this patient?
