# Supplementary material for: A longitudinal blended learning curriculum for bedside ultrasound education in pulmonary and critical care fellowship
Source: BMC Med Educ. 2025 Jan 24;25:123. doi: 10.1186/s12909-024-06584-8 (PMC11762126; doi:10.1186/s12909-024-06584-8)
Supplement: Supplementary file 6 — Additional file 6: OSCE Videos [file 12909_2024_6584_MOESM6_ESM.pptx]

## Slide 1
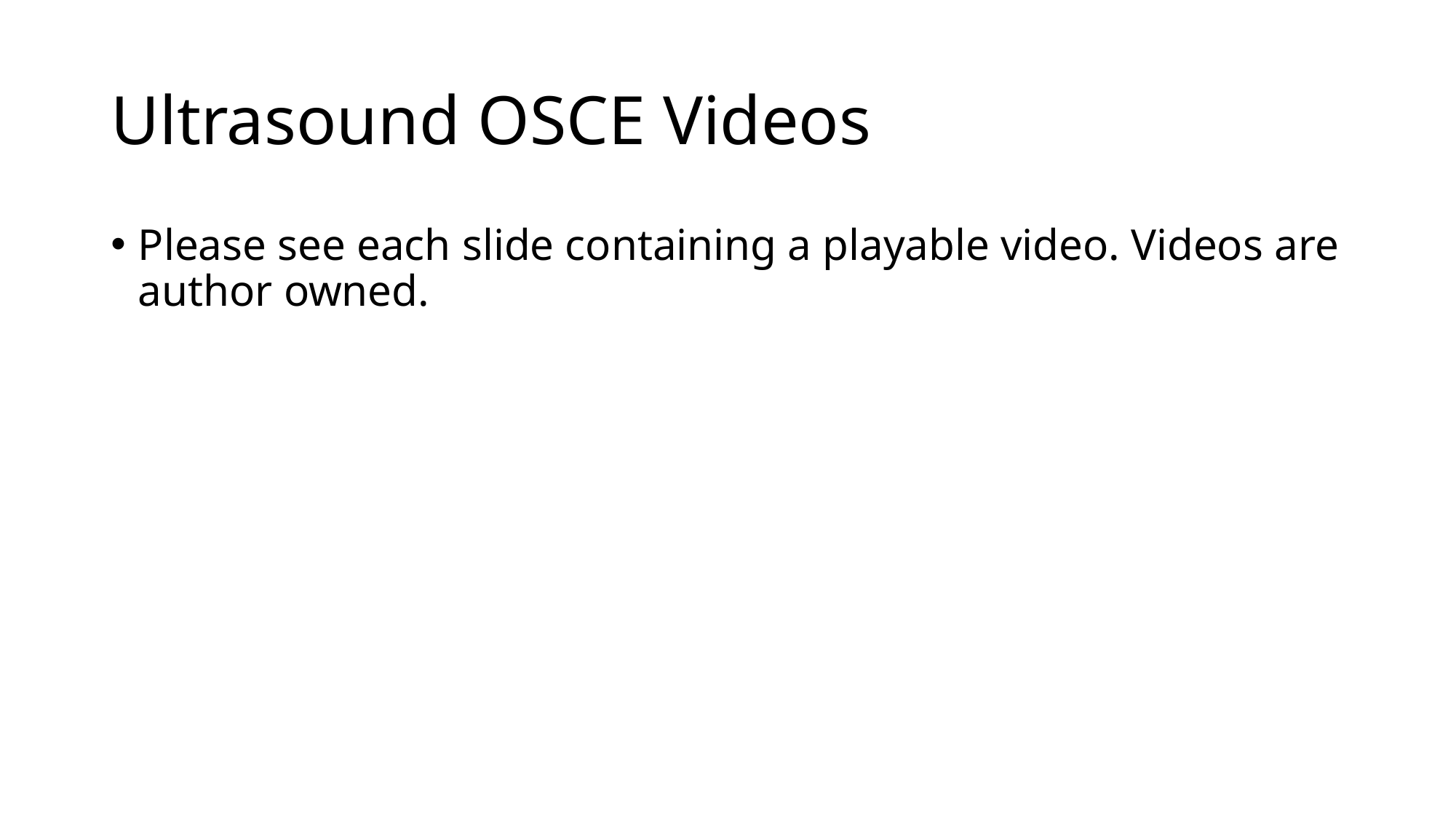

# Ultrasound OSCE Videos
Please see each slide containing a playable video. Videos are author owned.

## Slide 2
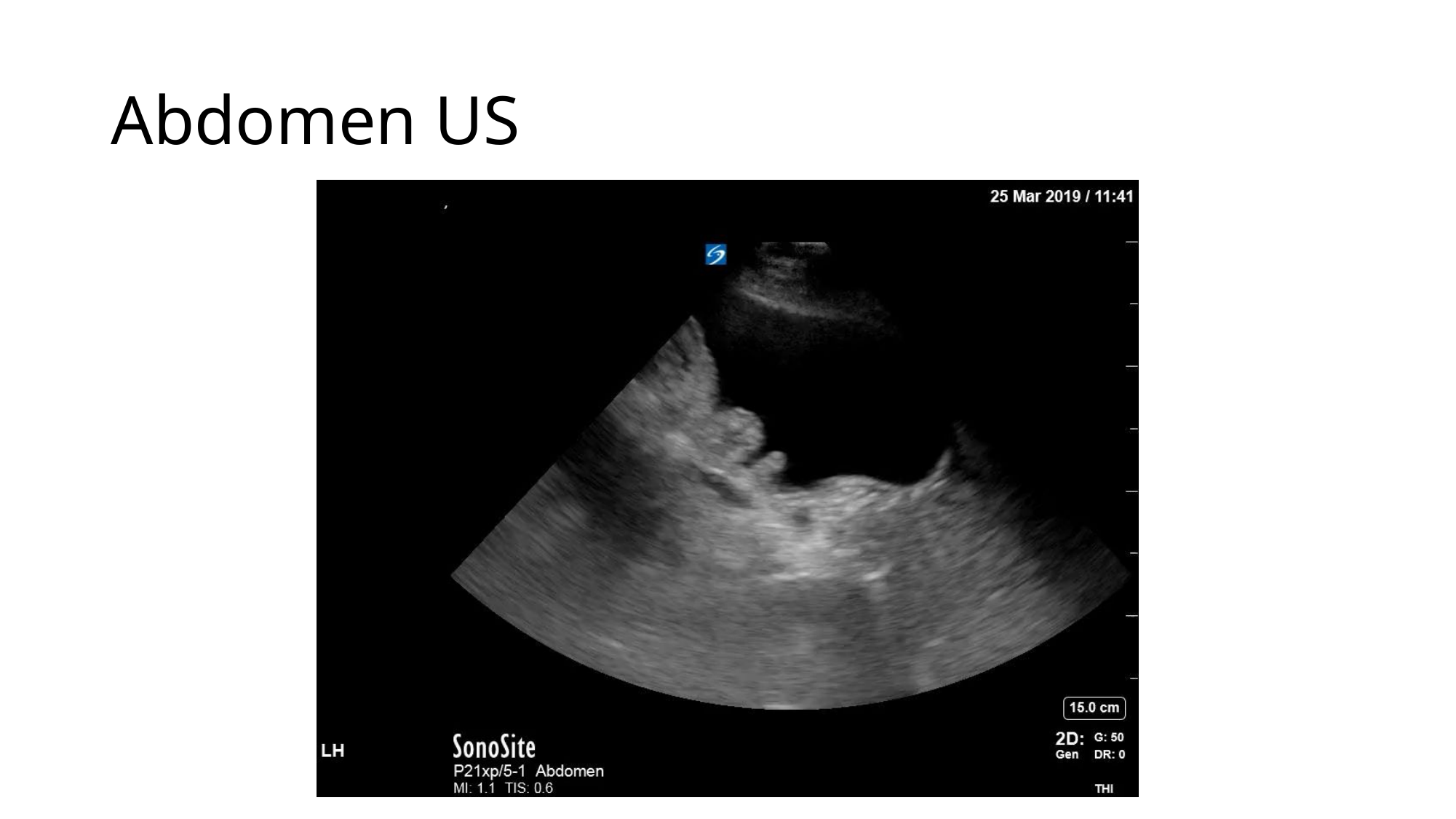

# Abdomen US

## Slide 3
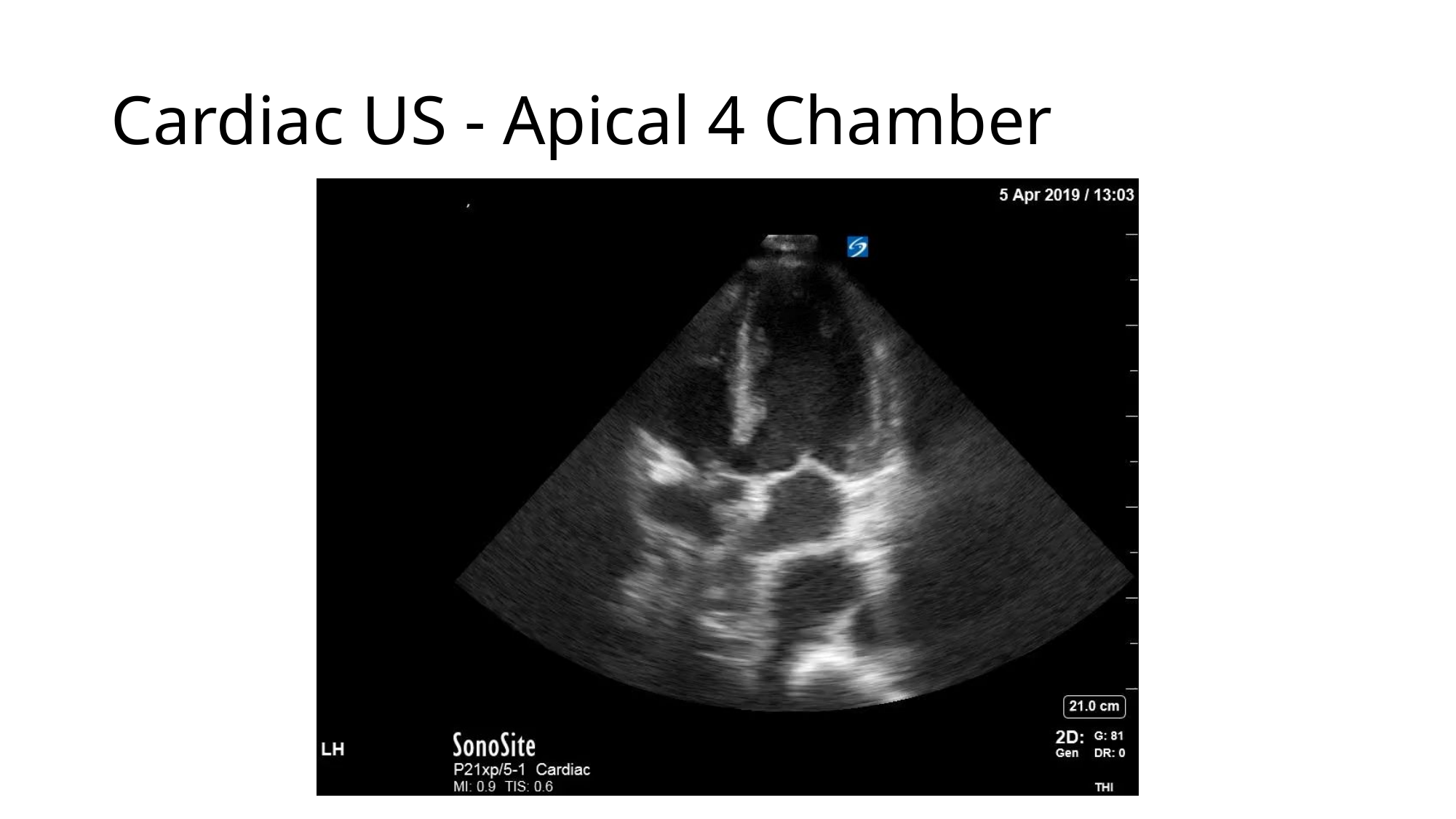

# Cardiac US - Apical 4 Chamber

## Slide 4
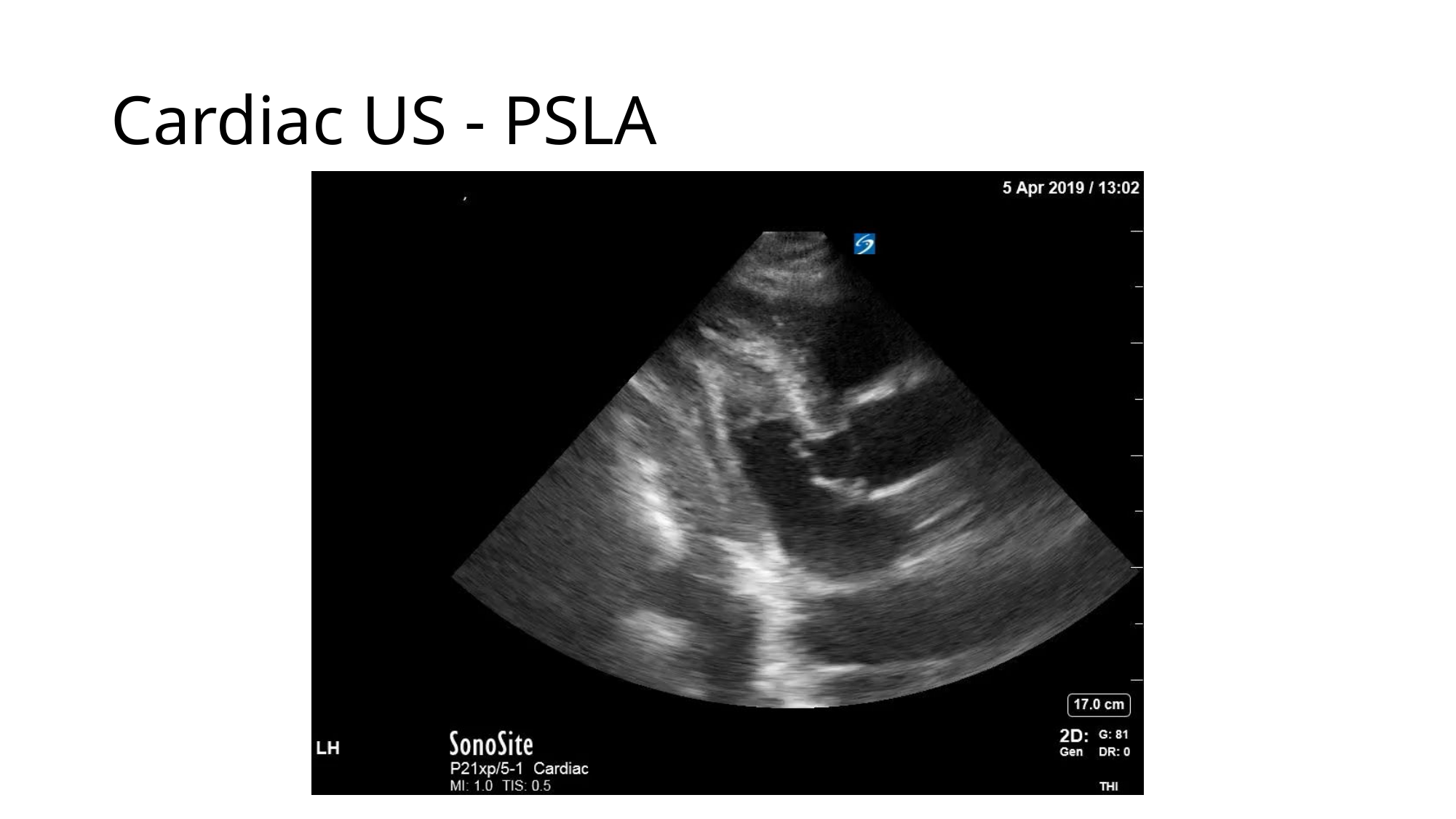

# Cardiac US - PSLA

## Slide 5
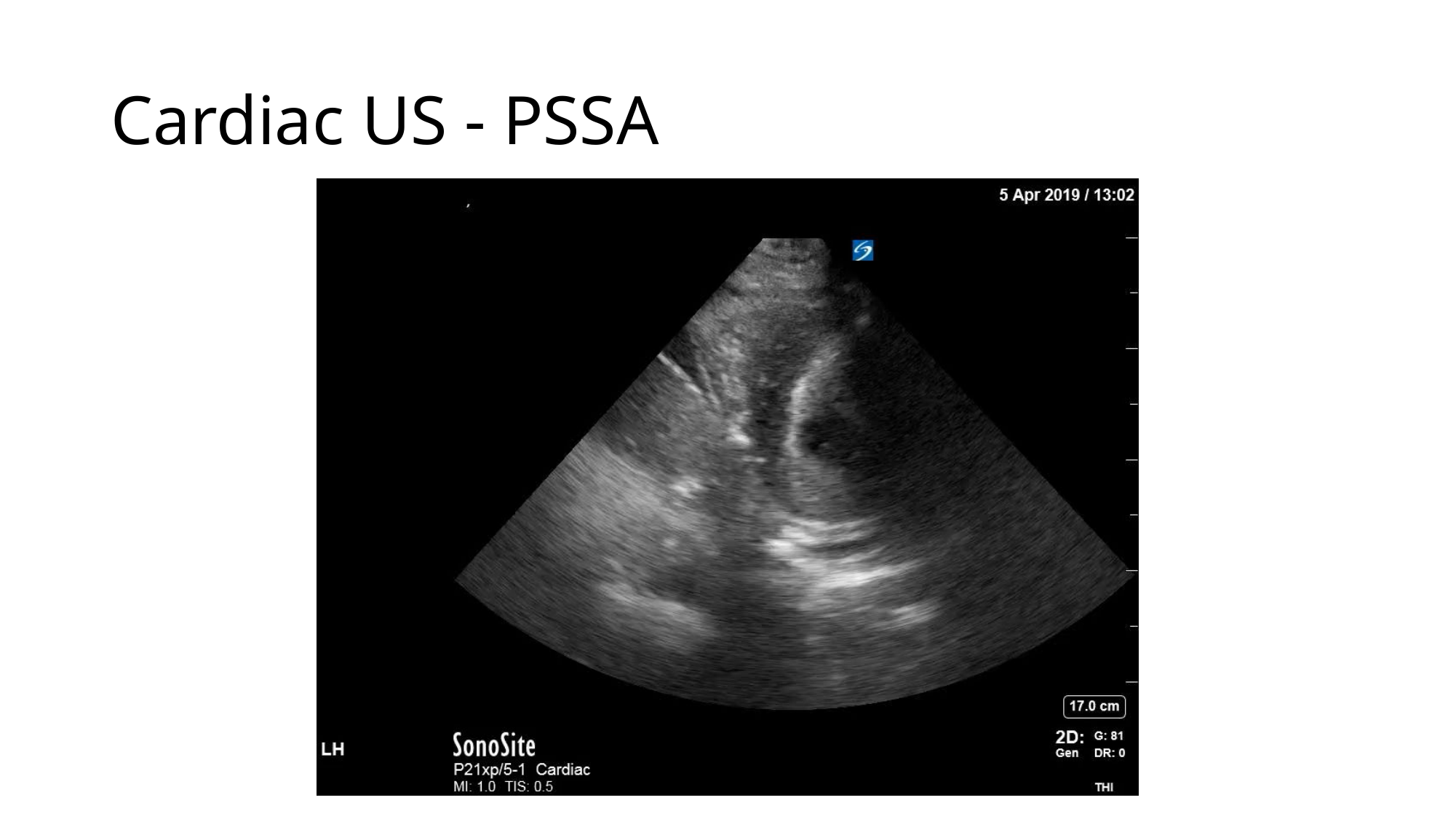

# Cardiac US - PSSA

## Slide 6
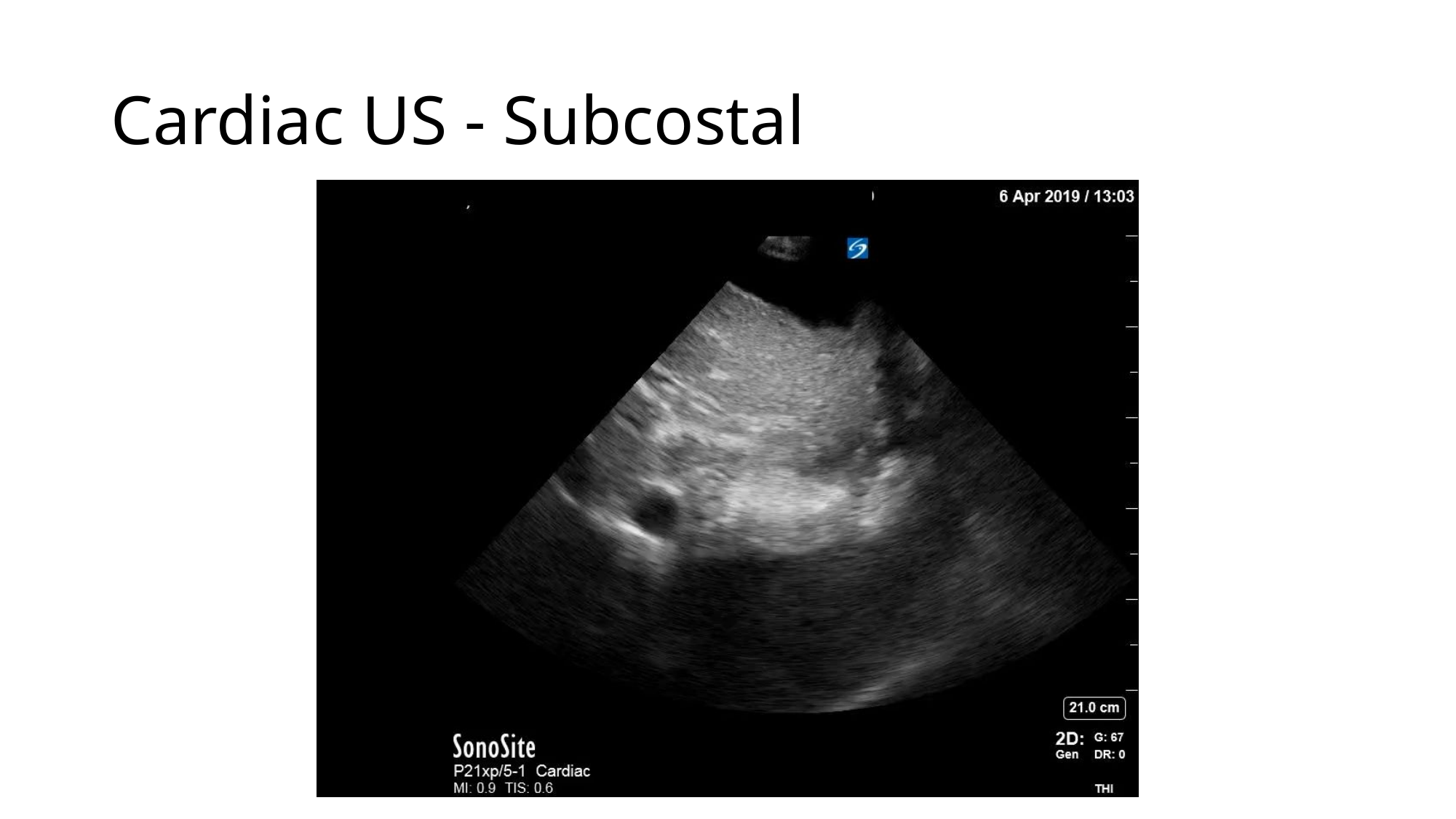

# Cardiac US - Subcostal

## Slide 7
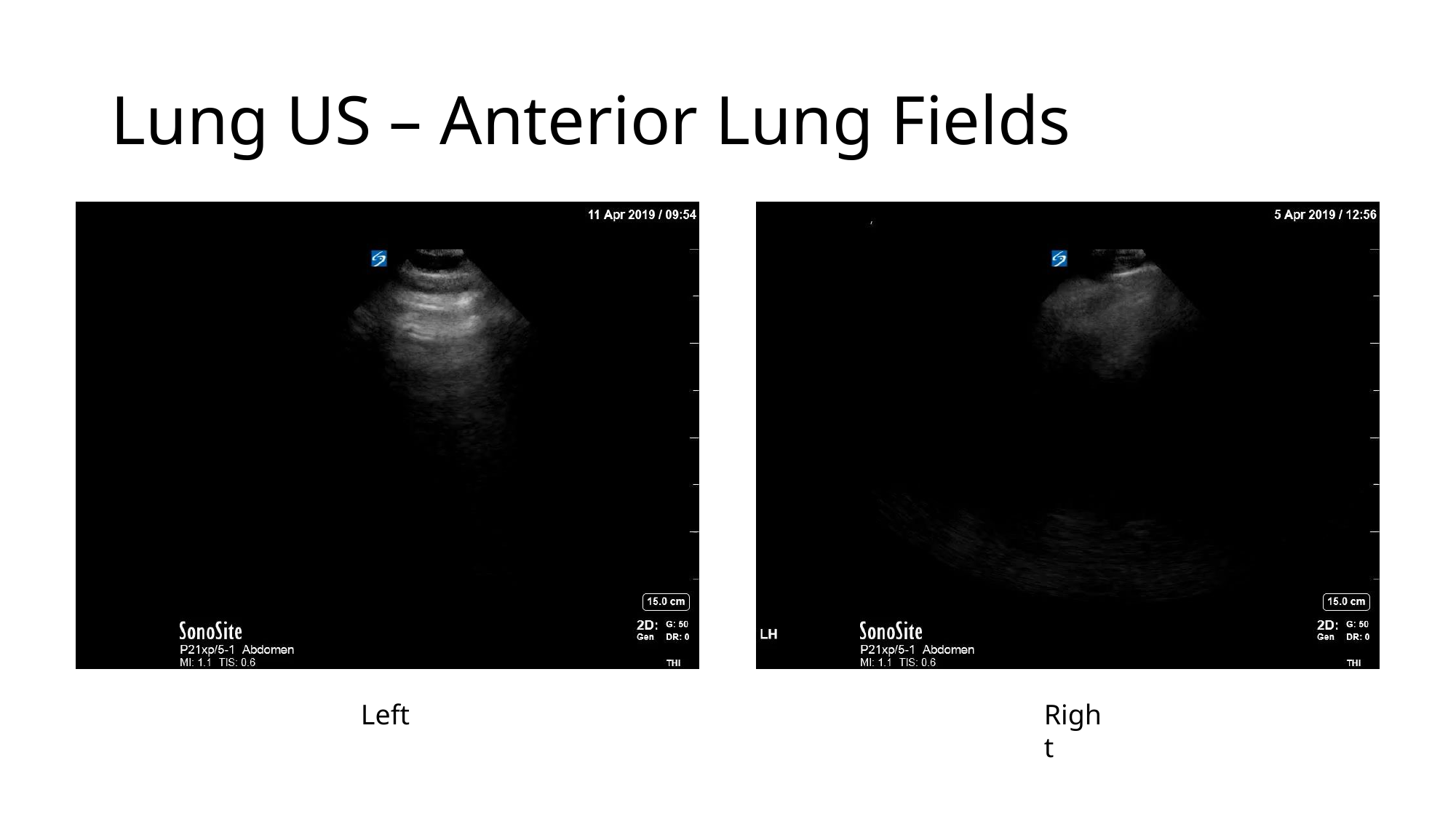

# Lung US – Anterior Lung Fields
Right
Left

## Slide 8
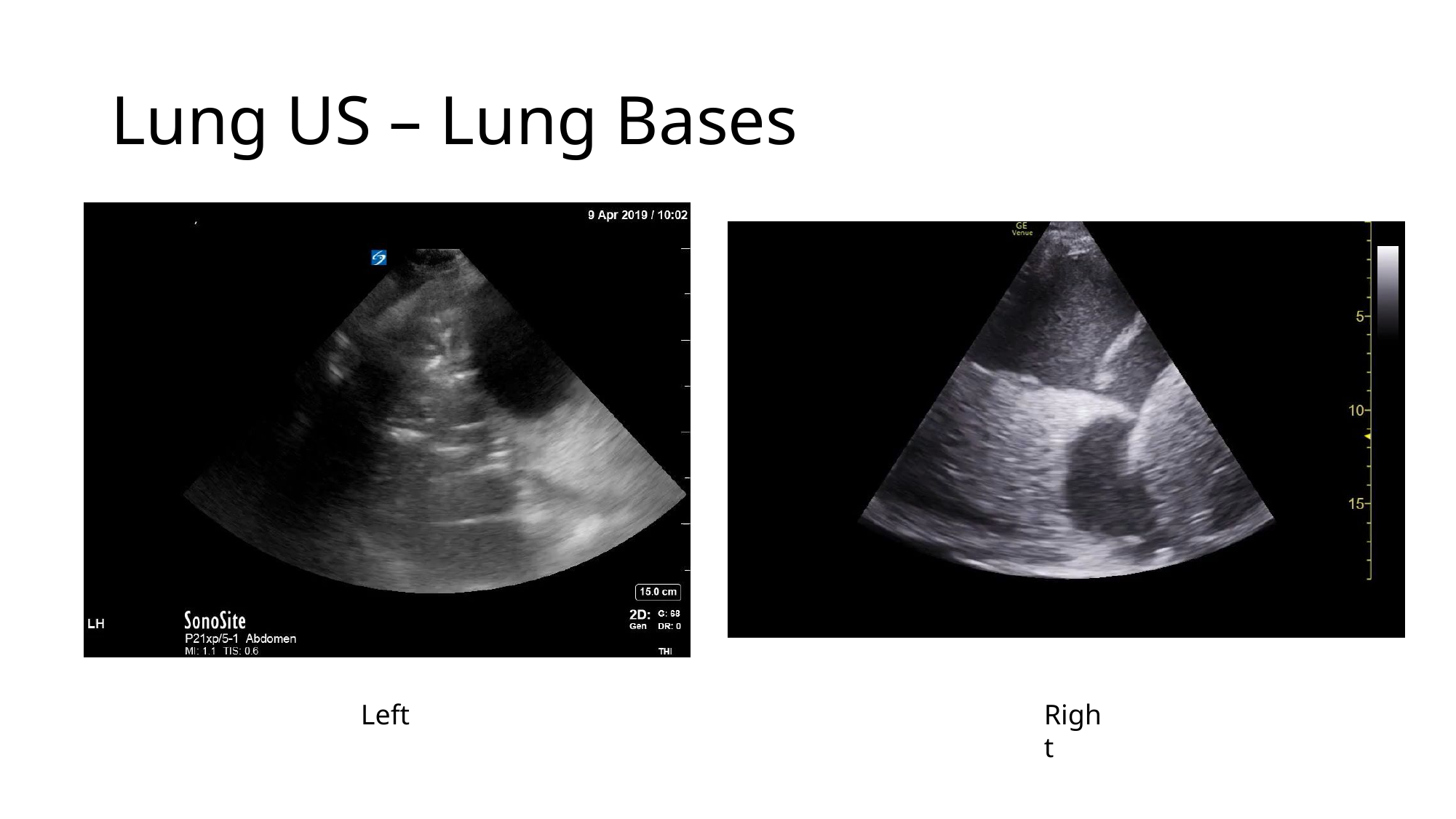

# Lung US – Lung Bases
Right
Left
