# Supplementary material for: A longitudinal blended learning curriculum for bedside ultrasound education in pulmonary and critical care fellowship
Source: BMC Med Educ. 2025 Jan 24;25:123. doi: 10.1186/s12909-024-06584-8 (PMC11762126; doi:10.1186/s12909-024-06584-8)
Supplement: Supplementary file 7 — Additional file 7: OSCE Key [file 12909_2024_6584_MOESM7_ESM.docx]

**Ultrasound OSCE Answer Key**

**Exam:** Limited chest, Limited ECHO, Limited abdomen

**Indication:** Shock state, Respiratory distress

**Findings:**

Lung US:

R anterior b-lines with effusion

L anterior lung field: normal aeration pattern [lung sliding and a-line pattern]

R base: large sedimented pleural effusion, complex; w/ consolidative lung pattern

L base: consolidative pattern

ECHO:

hyperdynamic LV

no pericardial effusion

normal RV size and function

IVC poor visualization

Abdomen:

Simple appearing moderate ascites

Interpretation:

The cause of shock state is likely combination of hemorrhagic [R hemothorax] and septic shock [L PNA].

The cause of respiratory failure is likely R hemothorax along with L PNA at the base.

**What would be your immediate management for this patient?**

Intubate

Resuscitation with pack RBCs

R diagnostic thoracentesis and/or chest tube [some may say CT surgery, but should perform a diagnostic thoracentesis first]

Possible diagnostic paracentesis

Antibiotics

Pressors as needed
